# Physiological, Metabolic, and Mitochondrial Adaptations to a One-Week Endurance Training Camp in Recreational Athletes: An Observational Study

**DOI:** 10.3390/sports14050200

**Published:** 2026-05-13

**Authors:** Daniel Alexander Bizjak, Lucas John, Moritz Munk, Marie Reiter, Nea Lüders, Johannes Kirsten, Alexander-Stephan Henze, Sebastian Viktor Waldemar Schulz

**Affiliations:** 1 Division of Sports and Rehabilitation Medicine, Department of Internal Medicine, University Hospital Ulm, 89075 Ulm, Germany; lucas.john@uniklinik-ulm.de (L.J.); moritz.munk@uni-ulm.de (M.M.); reiter.marie@icloud.com (M.R.); nea.lueders@uni-ulm.de (N.L.); johannes.kirsten@uniklinik-ulm.de (J.K.); alexander-stephan.henze@uniklinik-ulm.de (A.-S.H.); sebastian.schulz@uniklinik-ulm.de (S.V.W.S.); 2 Institute of Medical Engineering and Mechatronics, Ulm University of Applied Sciences, 89081 Ulm, Germany

**Keywords:** training camps, endurance training, metabolic adaptation, stress, mitochondrial adaptation, sleep, nutrition

## Abstract

Endurance training camps are well established in elite sports, but one-week camps for recreational endurance athletes have recently gained popularity despite limited scientific evidence. This study investigated the effects of a one-week endurance training camp on body composition, endurance performance, and markers of metabolic stress and mitochondrial adaptation in recreational athletes. Female and male endurance athletes (≥18 years) participated in a professionally guided one-week endurance training camp. Assessments included body composition, running diagnostics, sleep-quality/recovery-stress questionnaires, nutrition/energy balance diaries, blood profiling, and mitochondrial biogenesis markers. Measurements were conducted before (pre), during (camp), and after the camp (post). A total of 35 participants (18 male/17 female) were included. Body mass and body fat decreased from pre- to post-camp. Lactate concentrations at threshold levels changed, while velocities at fixed lactate concentrations and maximal oxygen uptake did not significantly improve. Post-camp, lactate dehydrogenase, klotho, and vitamin D increased, whereas interferon-γ, kynurenine, cortisol, creatinine, and ferritin decreased. Plasma mitochondrial and nuclear DNA abundance, as well as PGC1-α expression, increased, while vascular endothelial growth factor decreased. A one-week endurance training camp in a holiday-like setting induces measurable physiological, metabolic, and mitochondrial adaptations in recreational athletes and is associated with reduced systemic and psychological stress. However, the concurrent increase in muscle- and cell-stress markers indicates a substantial physiological load.

## 1. Introduction

The basic prerequisite for training-adaptive processes is a continuous, structured and periodized endurance program geared towards performance and regeneration processes. This leads, cumulatively, to improvements in the cardiovascular system and optimization of metabolic and muscular processes. As a result, athletic fitness and performance increase [1]. In addition, endurance training has been shown to have positive effects on mental health [2,3] or sleep quality [4] in healthy individuals and numerous disease-affected populations.

Due to the increasing number of ambitious recreational athletes, the number of marketing agencies organizing (professional) running camps in combination with holiday atmosphere is also increasing. The proclaimed advertised benefits of these, usually one-week, training camps often include more effective and innovative training methods, optimization of running training and success, as well as improved performance (e.g., examples of marketing agencies in [5,6,7]). These advertising promises create high expectations among participants regarding the positive effects of a one-week training camp, even though the evidence-based principles are not known yet.

With regard to the acute and short-term effects of a training camp, comparisons can only be made with data from professional athletes—and only to a limited extent. As a rule, a training camp in competitive sport lasts longer and varies between 9 and 40 days in the majority of the available literature [8,9,10]. However, there is still a lack of data on the effects of a combination of “holiday trip and training camp” lasting only one week on stress, sleep and regeneration. In general, the feeling of recovery, regeneration and sleep quality increases during holiday trips in the general population [11], whereas no studies exist that systematically examine the effect of “classic” holidays on regeneration markers in endurance athletes. Approximations can only be made by looking at tapering, which is a structured reduction in training load (usually volume, sometimes intensity), often for 1–3 weeks before a major event [12], and usually involves reduced load, possibly more rest and lower stress [13], but lacks the holiday aspect.

Continuous moderate or intensive endurance training with sufficient breaks can also have a positive effect on sleep quality [4,14], while non-functional high-intensity training over several days (such as at a training camp) can lead to a deterioration in sleep [15]. The combination of both—a holiday setting, which should decrease psychological stress, and increased training load, which exerts physiological stress—has not been examined in the current literature.

Examinations regarding the effects of training camps are mainly focused on elite athletes, which significantly limits the generalizability to other sports or recreational mass sports. In addition, especially in endurance sports, training camps are mainly held at different altitudes—usually at altitudes between 2000 and 3000 m—in order to stimulate hypoxia-induced hematopoiesis and thus increase oxygen transport capacity [16,17,18,19]. Further improvements may be induced by mitochondrial biogenesis [20] or increased mitochondrial oxidative enzyme activity [21], but these studies are limited to small participant numbers and several co-factors, like the distinction between altitude and training effects. In addition, these training modalities occur rather rarely in recreational sports, and data is therefore not available. Thus, while the positive effect has been shown in professional athletes [8,9,10], there is still a lack of fully verifiable data for non-professional athletes.

For this reason, this observational study aimed to analyze the benefits and effects of a one-week endurance training camp for female and male participants of different performance levels on their physical and mental performance. The training camp investigated in this study promised participants “training groups for every level, strength and technique training, regenerative units (balance and relaxation with yoga and Pilates units), enjoy exciting lectures (from nutrition to training design to optimal running equipment), the Spanish spring sun, new friendships, good mood and motivation, as well as sports medical care on site” [7].

In addition to performance tests and biomarker analysis, questionnaires and training/nutrition protocols were applied to evaluate the effect on subjective and objective performance, stress and regeneration, as well as mitochondrial biogenesis, in a holiday-like setting combined with increased training load and professional supervision.

## 2. Materials and Methods

### 2.1. Study Aim, Design, and Setting

This prospective, observational study investigated the effects of a professionally guided one-week endurance training camp on body composition, endurance performance, and blood- and saliva-derived markers of metabolic stress status and mitochondrial function in recreational endurance athletes. Participants were recruited from an ASC training camp conducted from 1 March to 8 March 2025 in Mallorca, Spain. Pre-participation questionnaires regarding training volume and competition frequency, as well as pre-camp running performance tests, revealed that the study population consisted of Tier 1 to 2 athletes, as defined by McKay et al. (recreationally active and trained/developmental athletes, respectively) [22].

### 2.2. Study Population

Participants were eligible for inclusion if they (i) were aged ≥ 18 years and (ii) participated in the ASC training camp from 1 to 8 March 2025 in Mallorca, Spain. Participants were excluded if (i) they were unable to take part in the whole training camp due to pregnancy, injury or illness or (ii) if they did not comply to the study procedures.

### 2.3. Body Composition

Anthropometric measurements included height, body mass and body composition. Height was measured without shoes, in light clothing, with a standardized scale. For measuring body mass and body composition, a bio-impedance scale (InBody 770, InBody Europe B.V., Eschborn, Germany) was used. All participants were in a fasted state.

### 2.4. Nutrition and Training

The training camp consisted of up to five daily exercise sessions, including running, cycling, stability training, aqua jogging and yoga/pilates (see the overview in Supplementary File S1). All sessions were supervised by professional running and cycling coaches, who were professional athletes at the national level. There was neither an obligation to take part in all training sessions or programs offered by the organizer, nor any restrictions with regard to additional training or relaxation sessions (yoga, Pilates, etc.). All participants had to record all training sessions for determining total exercise duration and caloric expenditure. In addition, the intake of nutrients was tracked via the FDDB app. In the FDDB app, all participants had to track their intake of macro- and micronutrients. The app is based on the reference values for nutrient intake from the German Nutrition Society (DGE) and the respective individual profile data (age, gender, height and weight) [23]. The participants entered their daily food intake during the study period of three weeks. The nutrition diary started exactly one week before the first day of the training camp, and ended exactly one week after the last day of the training camp. At the end of the study period, all participants exported their nutrition logs for analysis of the consumed caloric, as well as macronutrient, intake by the study team.

### 2.5. Sleep Quality, Stress and Recovery

Furthermore, sleep quality was assessed using the validated German version of the Athlete Sleep Behavior Questionnaire (ASBQ), specifically designed for the sleep behavior of athletes (FSVS). The FSVS consists of 18 items on a scale from 1 = never to 5 = always, and was completed once a week each Sunday morning during the study period and filled out in writing [24].

In addition, the Short Recovery and Stress Scale (SRSS, German version) had to be performed daily via an online questionnaire. The SRSS is a standardized self-assessment procedure to assess the current recovery-stress state of an athlete on physical, mental, emotional, and overall levels, and includes the items (i) physical performance capability, (ii) mental performance capability, (iii) emotional balance, (iv) overall recovery, (v) muscular stress, (vi) lack of activation, (vii) negative emotional state and (viii) overall stress [25].

An overview of all study procedures at the respective time points is provided in the Supplementary Figure S1A.

The study was conducted in accordance with the Declaration of Helsinki and was approved by the local ethics committee (No. 413/24, Ulm University). After providing detailed information, written informed consent was obtained from all participants. This study is reported in accordance with the *Strengthening the Reporting of Observational Studies in Epidemiology* (STROBE) guidelines [26]. As this study was not designed as an intervention study and consisted of an observational approach, no pre-registration in a clinical database was conducted.

### 2.6. Endurance Performance

To determine the current training status of all participants, a lactate exercise test was performed 1–3 days before (pre-camp) and eight days after the training camp (post-camp) to allow sufficient regeneration time. The lactate diagnostics were performed on a 400-m running track by experienced professional sport scientists. In short, participants ran together in groups of 3–6 people, with the pace being continuously increased every three minutes by 2 km/h, beginning with 6 km/h, until subjective or objective exhaustion [27]. The target pace was acoustically signaled at predefined markers around the track, which had to be reached at the designated time. Otherwise, the respective participant was not allowed to enter the next pace stage. The same applied if the participant reached subjective total exhaustion despite strong verbal motivation. After each “pace stage”, heart rate was assessed and 20 µL capillary blood was taken from the ear lobe for lactate analysis (Biosen S-Line, EKF Diagnostics Barleben, Germany). Lactate at the lactate threshold (LT; the first increase in the lactate curve), lactate at the individual anaerobic threshold (IAT), velocity at maximum lactate (Lacmax), maximal oxygen capacity (VO_2_max) and heart rate (HR) at IAT were calculated to determine individual performance capabilities at both time points. As well as lactate analysis, VO_2_max was estimated using the Ergonizer software (version 5.16.2, Freiburg, Germany). The software does not rely on a single predictive equation but applies a multi-step computational approach based on exercise performance data. First, maximal power output (Pmax) was calculated from the final completed and partially completed stages of the incremental test using the following equation: Pmax = P_penultimate + (P_increment × t_last stage/t_stage). Based on the assumption of a constant mechanical efficiency, the corresponding energy expenditure was calculated for each workload. From this, oxygen demand was estimated and subsequently extrapolated to determine VO_2_max [28].

### 2.7. Metabolic Stress Status and Mitochondrial Function

#### 2.7.1. Sampling

Pre- and post-camp, blood and saliva samples were collected in a fasted state prior to the performance diagnostic to avoid acute exercise-induced confounding. Venous blood was drawn from the participants’ *vena mediana cubiti* (EDTA, S-MONOVETTE, Sarstedt, Nümbrecht, Germany) and immediately centrifuged at 2380 g for 10 min at room temperature. Plasma was aliquoted into 0.5 mL tubes and stored at −80 °C until final analysis.

A complete blood count (CBC) was performed by the medical laboratory of university hospital Ulm. Further variables included creatine kinase (CK), urea, creatinine, ferritin, bilirubin, aspartate transaminase (AST), alanine aminotransferase (ALT), gamma-glutamyltransferase (GGT), glucose, cholesterol, triglyceride, high-density lipoprotein (HDL), low-density lipoprotein (LDL), glomerular filtration rate (GFR), vitamin D, and thyroid-stimulating hormone (TSH).

For saliva sampling, all participants had to drool 5 mL of saliva into a 50 mL tube (Fisher Scientific, Schwerte, Germany). Samples were immediately centrifuged at 2380 g for 10 min at room temperature. The clear supernatant was aliquoted into 2 mL tubes and stored at −80 °C until analysis.

#### 2.7.2. Markers

For assessing molecular stress as well as cell/mitochondrial adaptation, kynurenine, lactate dehydrogenase (LDH) (Thermo Fisher Scientific, Darmstadt, Germany), interferon-γ (IFN-γ) (MyBiosource, San Diego, CA, USA), klotho (Cloud-Clone Corp., Wuhan, China), vascular endothelial growth factor (VEGF) (Thermo Fisher Scientific, Darmstadt, Germany), growth differentiation factor 15 (GDF-15) (Thermo Fisher Scientific, Darmstadt, Germany), mitochondrial DNA (mtDNA), and PGC1-alpha-DNA were measured.

MtDNA, nuclear gene B2M and PGC1-alpha were measured via real-time qPCR (RT-qPCR) analogous to established protocols [29], with GAPDH as the established reference gene for endurance exercise [29,30]. Primers for mtDNA (hMitoF3: CACTTTCCACACAGACATCA, hMitoR3:TGGTTAGGCTGGTGTTAGGG) and B2M (hB2MF1: TGTTCCTGCTGGGTAGCTCT; hB2MR1: CCTCCATGATGCTGCTTACA) were designed using GeneGlobe and ordered together with PGC1-alpha primers from QIAGEN (Hilden, Germany). Relative gene quantification was determined by established protocols [31,32]. All samples were analyzed in duplicates.

Cellular stress and adaptation markers LDH (Thermo Fisher Scientific, Darmstadt, Germany), IFN-g (MyBiosource, San Diego, CA, USA) and klotho (Cloud-Clone Corp., Wuhan, China), as well as markers for (mitochondrial) biogenesis—VEGF (Thermo Fisher Scientific, Darmstadt, Germany) and GDF-15 (Thermo Fisher Scientific, Darmstadt, Germany)—were measured by ELISA according to the manufacturers’ instructions.

Plasma kynurenine concentrations were determined by spectrometry using an established protocol [33]. In summary, the plasma samples were subjected to a process of deproteinization using acetic acid trichloride. Following this procedure, the stable metabolite kynurenine reacted with 4-dimethylamino-benzaldehyde (Ehrlich’s reagent) to form a yellow product. This reagent is used in the detection of primary amino groups, pyrrole and indole derivatives. Absorption was measured at a wavelength of 492 nm in a linear scale from a 0.5 to 100 μM concentration of N-formylkynurenine, proportional to the activity of the enzyme indoleamine-2,3-dioxygenase, and quantified using a standard curve prepared with known concentrations of the product.

Concentrations of cortisol, C-reactive protein (CRP) and uric acid were assessed in saliva. All measurements were performed according to the manufacturer’s instruction (Salimetrics, Carlsbad, CA, USA).

### 2.8. Statistics

Data were analyzed using GraphPad Prism 11.1 (San Diego, CA, USA). Normality was assessed with the Kolmogorov–Smirnov test.

For paired comparisons of pre- and post-camp data, two-tailed *t*-tests were used for normal distributed data, and Wilcoxon matched-pairs signed-rank tests were applied otherwise. Effect sizes for normally distributed data are reported as Cohen’s d, where d > 0.2 signifies small, d > 0.5 medium, and d > 0.8 large effects for parametric tests, and for non-parametric testing, the Wilcoxon effect size r was used (effect: 0.10–0.3 (small), 0.30–0.5 (moderate), and > 0.5 (large)).

One-way ANOVA with the Holm–Šídák test and Greenhouse–Geisser correction (normal data) or Kruskal–Wallis with Dunn’s test (non-normal data) was used to analyze nutrition, FSVS and SRSS, as these questionnaires covered all weeks of the study period (pre-camp, training camp, post-camp). Effect sizes are reported as partial eta squared ($\eta_{p}^{2}$), where $\eta_{p}^{2}$ = 0.01 signifies small, 0.06 $\eta_{p}^{2}$ medium, and 0.14 $\eta_{p}^{2}$ large effects. Data are shown as mean ± SD unless noted; significance was set at *p* ≤ 0.05. For RT-PCR analysis (MITO, B2M, PGC1-alpha), only relative fold-change pre- and post-camp without additional testing can be reported.

An a priori power analysis was conducted using G*Power (version 3.1.9.7; Heinrich Heine University Düsseldorf, Germany) to determine the required sample size for detecting within-subject changes over the course of the one-week endurance training camp. Given the study design, a paired-samples *t*-test (two-tailed) was selected for primary analyses comparing pre- and post-camp outcomes.

The significance level was set at α = 0.05, with a desired statistical power of 1−β = 0.80. As no directly comparable studies investigating short-term training camps in recreational athletes were available, effect size assumptions were based on established conventions and the related exercise intervention literature [34].

As the examination of the “holiday effect” on mood state and stress was regarded as primary outcomes, moderate to large effects were expected for psychophysiological stress parameters due to the combined impact of structured training, environmental change, and group dynamics (Cohen’s d = 0.5–0.8). Based on these assumptions, the required sample size ranged between n = 15 and n = 34 participants.

As for the secondary regarded performance-related outcomes (i.e., maximal oxygen uptake [VO_2_max] and lactate metabolism) due to the short training camp duration, only small effects were anticipated (Cohen’s d = 0.2–0.3), as substantial physiological adaptations are unlikely to occur within a one-week training camp. Detecting such small effects would require large sample sizes (n ≈ 90–200), which was not feasible within the present study framework. Therefore, analyses of performance outcomes were considered exploratory.

To ensure adequate statistical power for the primary outcomes related to mental and physiological stress, while accounting for potential dropouts and inter-individual variability, a target sample size of at least 34 participants was defined. This sample size was deemed sufficient to detect moderate effects with adequate power in within-subject comparisons.

The power analysis followed established statistical guidelines for behavioral sciences and experimental research [34].

In an exploratory approach, possible sex differences in laboratory parameters were examined. One-way ANOVA with the Holm–Šídák test and Greenhouse–Geisser correction (normal data) or the Kruskal–Wallis test with Dunn’s test (non-normal data) was used to analyze subgroup differences between women (n = 17) and men (n = 18).

## 3. Results

### 3.1. Body Composition

From initially 41 screened participants, 6 dropped out due to injury/disease or organizational reasons. Thus, 35 (49% female) endurance athletes participated in this study. One further drop-out occurred due to injury before post-testing. Seven BIAs could not be conducted due to measurement failures of the BIA scale at the end of the study, resulting in twenty-seven BIA measurements post-camp. A participant flow diagram is provided in Figure S1B.

The participants showed a significant decrease in body mass (*p* = 0.0306; $\eta_{p}^{2}$ = 0.133), BMI (*p* = 0.0174), and body fat (*p* = 0.0370; $\eta_{p}^{2}$ = 0.122). The phase angle increased from pre- to post-training camp (*p* = 0.0003) with high effect ($\eta_{p}^{2}$ = 0.419). No change was observed for skeletal muscle mass, whereas the phase angle increased (*p* = 0.0003) (Table 1).

### 3.2. Nutrition and Training

Regarding macronutrient intake and distribution, an increase in fat ($\eta_{p}^{2}$ = 0.4620), carbohydrates ($\eta_{p}^{2}$ = 0.4345) as well as protein ($\eta_{p}^{2}$ = 0.4388) could be observed from the pre-camp stage to the training camp (all *p* < 0.001), followed by a decrease post-camp (*p* < 0.001 (Supplementary Figure S2A)). There was no difference in relative macronutrient distribution during the whole study period. Caloric intake was significantly increased and adapted to energy expenditure during the training camp where training amounted to approximately three hours for runners and five hours for cyclists per day, respectively. This meant a three-fold increase in training volume compared to the pre- and post-camp periods during the camp. Accordingly, the number of overall training sessions (including running, cycling and stability training, as well as other exercise activities) recorded by the participants increased significantly from 6.1 ± 2.3 trainings per week pre-camp to 8.9 ± 3.4 (*p* = 0.0143) during the camp, before decreasing to 5.1 ± 2.3 post-camp (*p* = <0.0001) (Supplementary Figure S2). Calculating the mean difference in energy expenditure–energy intake during all weeks, no difference could be observed between the different weeks.

### 3.3. Endurance Performance

Compared to pre-camp, the participants showed changed lactate characteristics at the lactate threshold (*p* = 0.0193; $\eta_{p}^{2}$ = 0.159) and the IAT (*p* = 0.0185), as well as a higher heart rate at the IAT (*p* = 0.0158), with high effects for lactate ($\eta_{p}^{2}$ = 0.166) and HR at IAT ($\eta_{p}^{2}$ = 0.169), respectively. A trend for increased values and a medium effect could be detected for VO_2_max (*p* = 0.0551; $\eta_{p}^{2}$ = 0.107) (Table 2).

### 3.4. Sleep Quality, Stress and Recovery

Sleep quality scores did not change during the whole study period with scores between 34 and 35, representing good sleep quality over all weeks (<37 good; 37–41 medium; >41 bad; Supplementary Figure S3).

In general, the SRSS assessed items showed an increase in muscular stress score during the training camp with a concomitant decrease in lack of activation and negative emotional state scores. Despite an increase in overall stress and decreased overall recovery during the training camp, physical and mental performance capability remained stable during all weeks. Overall stress decreased post-camp compared to pre-camp values.

In detail, mean physical and mental performance capability decreased by 8.0% and 5.0%, respectively, during the camp, but increased by 7.4% and 5.2% compared to pre-camp values. These changes were accompanied by a decreased mean overall recovery of 14.3%, and a 53.8% increase in muscular stress. Conversely, mean lack of activation decreased by 11.0% with a concomitant 33.8% reduction in negative emotional states during the camp and 32.2% post-camp compared to pre-camp values. Emotional balance increased by 6% during the camp and by further 4.5% post-training-camp. Overall stress increased by 12% during the camp but decreased by 22.6% after the camp (Supplementary Figure S4 and Table S1).

### 3.5. Metabolic Stress Status

**Figure 1:**
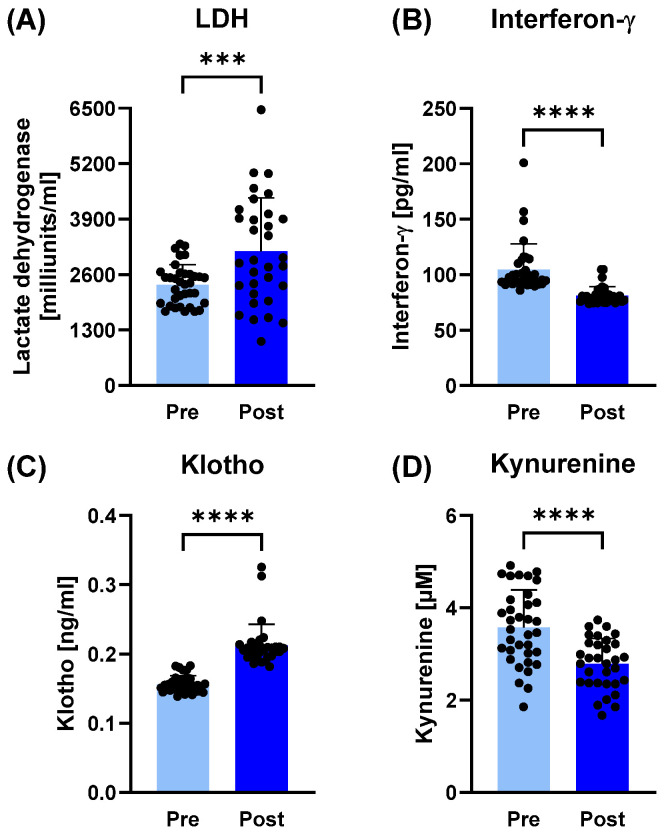
Plasma markers for (immune/cell) stress reactions before (pre) and after (post) the training camp. While (**A**) lactate dehydrogenase (LDH) and (**C**) klotho increased after the training camp (post), (**B**) interferon-γ and (**D**) kynurenine decreased after the training camp. *** *p* ≤ 0.001; **** *p* ≤ 0.0001.

**Figure 2:**
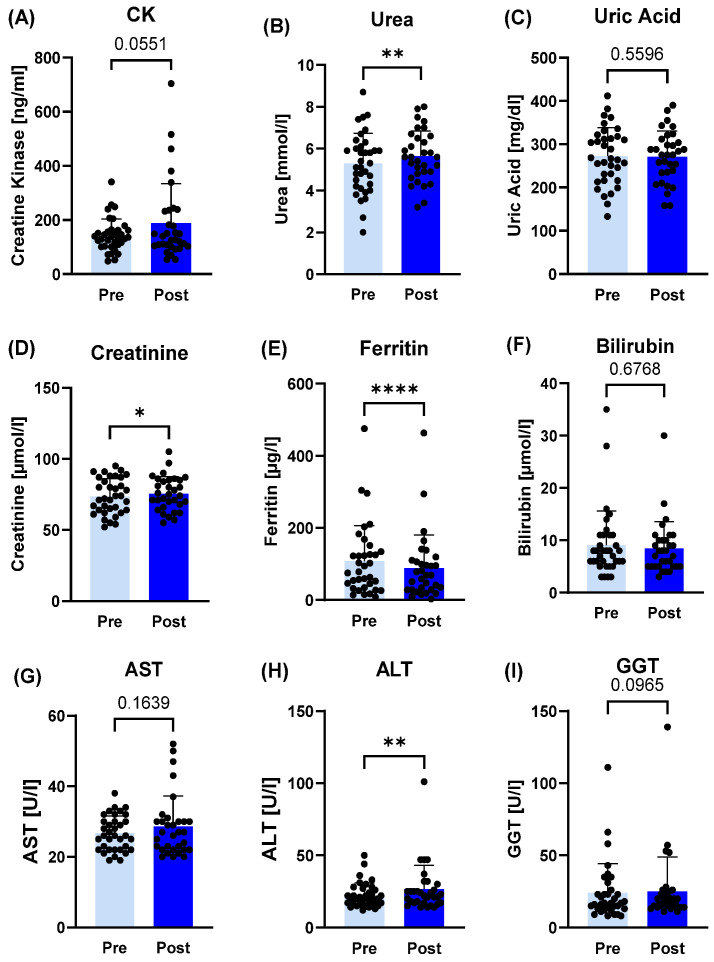
Plasma markers related immunological and cellular stress response before (pre) and after (post) the training camp. (**A**) Creatine kinase (CK), (**C**) uric acid, (**F**) bilirubin, (**G**) aspartate transaminase (AST) and (**I**) gamma-glutamyltransferase (GGT) did not change significantly post-camp, whereas (**B**) urea, (**D**) creatinine and (**H**) alanine aminotransferase (ALT) increased after the training camp. (**E**) Ferritin was the only biomarker that decreased post-camp. * *p* ≤ 0.05, ** *p* ≤ 0.01, **** *p* ≤ 0.0001.

In plasma samples, LDH (*p* < 0.001; d = 0.88), urea (*p* = 0.0074; r = 0.42), creatinine (*p* = 0.0183; r = 0.51), ALT (*p* = 0.0059; r = 0.48) and klotho (*p* < 0.0001; d = 1.76) significantly increased post-camp, while IFN-γ (*p* < 0.0001; d = 1.46), ferritin (*p* < 0.0001; r = 0.70) and kynurenine (*p* < 0.0001; d = 0.94) decreased post-training camp (Figure 1 and Figure 2).

**Figure 3:**
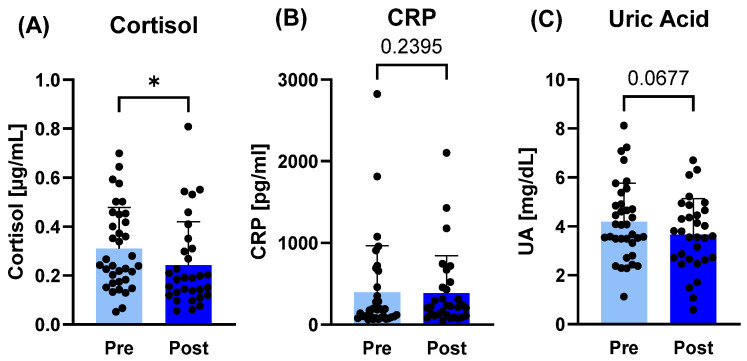
Salivary markers related to stress responses before (pre) and after (post) the training camp. (**A**) Cortisol concentrations decreased post- compared to pre-camp, whereas (**B**) C-reactive protein (CRP) and (**C**) uric acid (UA) concentrations remained unaltered. * *p* ≤ 0.05.

In saliva, only in cortisol concentrations was a difference observable, with decreased values post-camp (*p* = 0.0209; d = 0.40), whereas CRP and uric acid remained unaltered (Figure 3).

### 3.6. Mitochondrial Function and Vascular Proliferation

**Figure 4:**
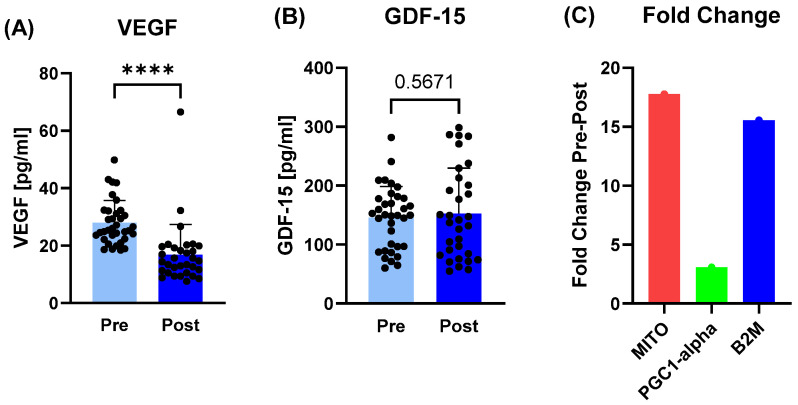
Markers related to mitochondrial biogenesis and proliferation before (pre) and after (post) the training camp. (**A**) Vascular endothelial growth factor (VEGF) concentration decreased post-intervention, while (**B**) growth differentiation factor 15 (GDF-15) remained unchanged. (**C**) Relative fold change in mitochondrial DNA (MITO) increased by 17.77 post-training-camp in the plasma samples, while PGC1-alpha increased by 3.07 and nuclear DNA (B2M) by 15.56, respectively. **** *p* ≤ 0.0001.

While VEGF protein concentration as a marker for vascular proliferation decreased from pre- to post-camp (*p* < 0.0001), GDF-15 as a marker for mitochondrial dysfunction did not change. Instead, the relative expression of mitochondrial DNA circulating in plasma increased 17.77-fold post- compared to pre-camp, as did the nuclear gene B2M (15.56-fold) and PGC1-alpha (3.07-fold) (Figure 4).

### 3.7. Blood Profiling

#### 3.7.1. Blood Cells

Regarding blood cell changes pre- to post-camp, thrombocytes (*p* = 0.0063), relative distribution width (RDW) (0.0143; r = 0.52), hematocrit (*p* = 0.0481; d = 0.36) and mean corpuscular volume (MCV) (*p* < 0.0001; d = 1.29) increased after the training camp. Decreases could be observed for mean corpuscular hemoglobin concentration (MCHC) (*p* < 0.0001; r = 0.64), while leukocytes, red blood cells (RBCs), mean corpuscular hemoglobin (MCH) and hemoglobin remained unchanged (Supplementary Figure S5).

#### 3.7.2. Lipid Metabolism

While there was an increase in observable cholesterol (*p* = 0.0093; d = 0.47), high-density lipoprotein (HDL) (*p* = 0.0181; r = 0.49) and low-density lipoprotein (LDL) (*p* = 0.0136; r = 0.53), triglycerides and glucose concentration in plasma did not change pre- to post-camp (Supplementary Figure S6).

#### 3.7.3. Glomerular Filtration Rate, Thyroid-Stimulating Hormone and Vitamin D

The glomerular filtration rate (GFR) decreased pre- to post-camp (*p* = 0.0139) with a moderate effect (r = 0.43), while vitamin D concentrations increased significantly after the training camp (*p* < 0.0001; d = 0.89). Thyroid-stimulating hormone (TSH) levels did not change significantly (Supplementary Figure S7).

### 3.8. Sex-Stratified Subanalysis

Differences between women and men were detected with HDL (higher values in females pre- and post-camp), LDH (only increasing in females), kynurenine (only decreasing in males), creatinine (higher values in males pre- and post-camp), plasma uric acid (higher values in males pre- and post-camp), ferritin (higher values pre-camp in females), and higher values pre- and post-camp in males regarding RBCs, hemoglobin and hematocrit. MCHC only changed in females pre- to post-camp, but were higher in males post-training-camp (see detailed analysis in Supplementary File S2).

## 4. Discussion

This study is the first to systematically examine the effects of a one-week endurance training camp performed in a “holiday-like” setting on physiological performance, anthropometry, stress and regeneration markers, sleep behavior, and biomarkers related to mitochondrial biogenesis in non-professional endurance athletes. Although the augmented training volume and intensity resulted in marked elevations in exercise-related damage markers, namely LDH and a trend in CK, in addition to alterations in ALT and lipid metabolism, the subjective stress levels and cortisol decreased, similar to inflammation parameters. Despite the very short intervention period, measurable adaptations occurred at several physiological levels, supporting the concept that even brief training camps may induce relevant biological changes if the training load is sufficiently increased, with concomitantly positive effects on perceived stress levels in the holiday setting.

### 4.1. Body Composition and Endurance Performance

From a sports science perspective, it would not be common advice for athletes to undergo a week-long high-volume training camp at sea level in order to substantially improve endurance performance. In line with this, our lactate-derived performance outcomes (velocity at fixed lactate concentrations and threshold velocities) remained unchanged from pre- to post-camp, while several biomarkers indicated physiological and cellular-stress-related responses to the increased training load.

Despite statistically significant changes in lactate concentrations at LT and IAT, velocity-based performance indicators did not improve: velocities at fixed lactate concentrations (2, 3, and 4 mmol/L) remained unchanged from pre- to post-camp, and VO_2_max showed no significant change (trend only). Therefore, the observed shifts in lactate concentrations should not be interpreted as evidence of improved endurance performance, as absolute lactate concentrations show substantial biological variability and threshold-related performance is more appropriately reflected by the corresponding running velocity or power output [35]. Instead, the present pattern may reflect short-term alterations in lactate appearance/clearance and day-to-day variability under field conditions, while meaningful performance adaptations likely require a longer intervention period and/or more targeted training stimuli [35,36].

In contrast to high-intensity multi-session camps in collegiate runners—where adverse vascular effects such as increased arterial stiffness are frequently observed [19]—our study population did not exhibit detrimental cardiovascular markers, likely because the training was short-term, mixed-modality and voluntarily distributed across running, cycling, and alternative sessions such as aqua jogging and yoga. This suggests that the applied training load, although approximately three-fold higher than a normal training week, did not induce pathological overreaching in most participants.

Measurement of uncertainty and practical relevance should be considered when interpreting small pre- to post-camp differences in field-based outcomes. We observed small changes in body composition parameters (body mass/body fat) and an increase in phase angle from pre- to post-camp. Bioelectrical-impedance-derived body composition and phase angle can be influenced by hydration status and recent dietary intake, and prediction errors may be relevant when absolute changes are small [37,38]. Likewise, incremental field lactate testing is influenced by biological day-to-day variability as well as sampling/analytical variation, and changes in absolute lactate concentrations should not be equated with meaningful performance adaptations when velocity-based outcomes remain unchanged [35]. Reporting and interpreting effects in the context of absolute differences and measurement variability is essential in real-world training camp studies [39,40].

### 4.2. Nutrition

Dietary intake recommendations should take other factors into consideration including the actual training load, total energy expenditure, and body composition goals of the athlete [41]. Numerous athletes encounter challenges in meeting their energy and carbohydrate needs during a training camp. Moreover, maintaining adequate energy availability may assist in preventing overreaching and sustaining performance throughout periods of intense training [42]. Our participants increased their daily training volume three-fold to three hours in runners and five hours in cyclists, with concomitantly increased numbers of training sessions to approximately nine different exercise activities per day. Interestingly, despite this higher volume and intensity with less regeneration time between exercise session, their caloric intake during the camp increased accordingly while keeping macronutrient distribution consistent. This is consistent with findings from junior triathletes, who adapted their carbohydrate intake relative to the intensity and volume during different training phases: unfortunately, only measures of carbohydrate intake, not total caloric intake, protein, or fat were recorded. The study reports higher absolute carbohydrate intake in the intensive period (9.0 vs. 7.8 g/kg/day) but does not explicitly present the macronutrient ratios (percentage of total energy) [40]. It is noteworthy that the practice of nutrition monitoring during training-camp studies in professional athletes is infrequent, and when it occurs, it is seldom documented. A study with young female cross-country skiers found that over half had insufficient energy availability (EA) and carbohydrate (CHO) intake during a five-day-long training camp [42]. Low EA and CHO were linked to symptoms of overreaching, such as reduced muscular performance and higher submaximal lactate/RPE ratios. An intervention study (anti-oxidative food) with elite athletes at a three-week-long high-altitude camp showed improved overall macro- and micronutrient composition in the athletes’ diets but only minimal impact on measured nutrition-related blood parameters [39]. The main cohort effect was increased energy and carbohydrate intake to meet altitude recommendations. Many studies emphasize carbohydrate needs of >7–10 g/kg/day during high-volume endurance phases [43], which aligns with the increased intake during the training camp in the present cohort. Importantly, increased caloric intake may have supported the absence of maladaptive stress responses (e.g., decreased cortisol and kynurenine), highlighting the importance of adequate fueling during training camps.

### 4.3. Sleep Quality, Stress and Recovery

Interestingly, sleep quality in our study population remained stable throughout the whole three weeks. Previous studies show that increased training volume, particularly without adequate recovery, can impair sleep [15], whereas tapering improves it [44]. A review of elite athletes that compared “home” baseline to training-camp recordings reported mean reductions in total sleeping time of about 85 min and a drop in sleep efficiency by 8 percentage points during training camps [45]. Our observations with unchanged sleep quality may reflect the “holiday effect,” where environmental novelty, relaxation, and the absence of psychosocial work stress improve perceived recovery even under increased physical loads [11]. Moreover, the daily structure—alternating training, relaxation sessions, and social activities—may have mitigated the sleep disturbances often seen in high-performance training environments. In addition to its effects on physical health, exercise is well known for boosting mood, which can lead to much better sleep quality, reduced stress and risk of overreaching if there is an adequate amount of rest and regeneration between training sessions [46]. The assessment of subjective stress and recovery by the SRSS questionnaire confirmed this observation. Despite higher training volume during the training camp compared to their normal training and subsequently muscular fatigue, our recreational athletes experienced less mental stress and had higher activity scores during and after the training camp, suggesting a beneficial effect of the “holiday setting” without obligatory training schedules and with peers in a relaxed environment and atmosphere.

### 4.4. Metabolic Stress Status

A notable finding is the reduction in cortisol, IFN-γ, ferritin, and kynurenine post-camp. Cortisol is classically elevated with acute high-intensity exercise in blood and saliva, especially during prolonged stress or insufficient recovery [47,48]. Yet, its reduction after the training camp suggests decreased systemic stress despite the high training volume, possibly reflecting psychological recovery in a holiday setting, consistent with improved mood states seen in similar studies on the effects of short training camps on cyclists and swimmers [49,50]. Furthermore, we observed significantly increased vitamin D levels after the camp. Although subject to potential confounding factors such as season, sunlight exposure, physical activity, BMI, and illness, accumulating evidence indicates that higher vitamin D concentrations due to sun exposure or supplementation may reduce cardiovascular disease risk factors [51]. These benefits include decreased cortisol/cortisone ratio, improvements in exercise performance [51] and higher sleep quality [52,53]. Thus, there may be an interdependence between the reduced cortisol levels and the increased vitamin D levels, especially as significant increases in vitamin D concentration can be achieved in short times with sufficient sun exposure [54]. Nevertheless, since both the training camp sessions and leisure outdoor activity were conducted in a constantly sunny environment during the camp duration, discriminating between the relative contributions of exercise and UV-B exposure effects on the vitamin D increase is difficult. As individual sun exposure behaviors (e.g., duration of exposure, clothing, and sunscreen use) were not systematically assessed, the observed increase in vitamin D may be interpreted as the combined effect of environmental and behavioral factors, as well as the increased exercise volume and intensity.

The observed decrease in kynurenine further indicates reduced systemic stress and inflammation. Exercise is known to modulate kynurenine metabolism via upregulation of kynurenine aminotransferases, increasing peripheral conversion to kynurenic acid and thereby reducing neurotoxic load [33]. The current decrease may therefore reflect an enhanced anti-inflammatory or neuroprotective metabolic shift.

Klotho is considered an “anti-aging” protein due to its anti-inflammatory and antioxidant properties [55], as well as its role in cellular pathways regarding energy, glucose, and phosphate metabolism [56]. Several studies observed increased klotho values after acute [56] or chronic [55] exercise, and also in athletes compared to non-athletes [57], identifying klotho as possible exerkine with beneficial effects [55]. We also measured higher values of klotho concentrations after the training camp, indicating adaptive effects even after seven days of high-load training volume.

In contrast, ferritin, which often reflects inflammation rather than iron status in physically active individuals, decreased after the training camp, aligning with observations that endurance athletes with higher training levels often show lower inflammatory biomarkers [58]. This suggests that, despite the increased training load, our participants did not develop a systemic inflammatory state; rather, inflammation may have decreased relative to baseline. This would be in line with our observation of unchanged plasma CRP and uric acid concentrations in saliva and plasma.

LDH and ALT increased significantly, whereas CK, AST, and GGT did not change. This is consistent with the known variability and individual responsiveness of muscle-derived enzymes during short-term endurance overload [9]. The increase in LDH likely reflects acute metabolic and muscle membrane stress, whereas the moderate ALT increase may relate to muscle—rather than hepatic—stress, as ALT is also expressed in skeletal muscle [59]. These results reinforce the necessity of monitoring muscle load in non-professional athletes, since training volumes during camps may exceed habitual loads and induce transient muscle-cell stress. In addition, LDH is also a sensitive marker of intravascular hemolysis, which is particularly relevant in running athletes due to foot-strike hemolysis [60,61]. In combination with the observed changes in hematological markers (increased MCV and RDW, as well as decreased RDW), this might show the first signs of increased erythrocytic syntheses after the unaccustomed exercise-induced hematologic stress. Unfortunately, we did not measure haptoglobin or reticulocytes, which would provide further insights into this possible mechanism.

The decrease in GFR likely resulted from increased creatinine and reduced hydration at the time of testing—a well-established phenomenon following intensified endurance training [58]. Importantly, the changes remained within clinically acceptable ranges.

Although total cholesterol, HDL, and LDL increased after the training camp, these increases may reflect acute physiological responses rather than adverse cardiometabolic changes. Previous research has shown that short-term intensive endurance exercise can transiently elevate circulating lipid concentrations, reflecting increased lipolysis and fatty acid mobilization during intensive endurance exercise, as well as short-term metabolic adaptations to acute exercise stimuli [62,63]. In addition, changes in dietary intake during training camps—such as increased energy or fat consumption—may further contribute to altered lipid profiles. Importantly, the concurrent rise in HDL cholesterol is generally considered a favorable adaptation associated with endurance training [63]. Therefore, the present findings probably represent short-term, exercise-induced modulation of lipid metabolism due to the training camp.

### 4.5. Mitochondrial Function and Vascular Proliferation

The increase in circulating mtDNA, nuclear DNA (B2M), and PGC1-α after the camp is particularly noteworthy. PGC1-α is the master regulator of mitochondrial biogenesis and is strongly induced by endurance exercise, especially by high-metabolic-stress stimuli [1,64]. Although increases in circulating DNA may reflect cellular turnover or stress, the concurrent, probably downstream, rise in PGC1-α supports an adaptive, rather than purely damage-related, interpretation.

The unchanged GDF-15 levels further reinforce this view. Although the biological function of ubiquitously expressed GDF-15 is not completely understood, its expression can be induced in response to stress, such as mitochondrial dysfunction, via upregulation of the activating transcription factor 4. Thus, GDF-15 is the currently one of the most reliable biomarkers of mitochondrial dysfunction in plasma samples [65]. Its stability suggests that mitochondrial stress did not exceed physiological thresholds in our participant population, despite the training load.

The decrease in VEGF was unexpected, as exercise—particularly endurance activities—typically increases VEGF expression, especially after acute bouts [21]. Possible explanations include the following: (1) timing of blood sampling: VEGF peaks within hours after exercise and may be suppressed after several days of reduced load [66]. (2) Reduced inflammatory drive post-camp, as VEGF is sensitive to inflammatory cytokine levels [67]. (3) Physiological redistribution, where plasma VEGF decreases despite local angiogenic signaling in muscle [68].

Thus, while VEGF plasma concentrations decreased, this does not preclude angiogenic adaptations at the tissue level. Unfortunately, further methods like laser Doppler or laser speckle imaging for blood flow measurements or contrast-enhanced ultrasound for regional perfusion quantification were not available to examine vascular adaptations on the cellular/muscular level.

### 4.6. Sex Differences

Due to known differences in the muscular, cellular and energy-related metabolism between men and women [69], we aimed to look at possible differences in the analyzed markers in response to the training camp. Although limited by reduced statistical power, our subgroup analysis with female and male participants revealed distinct physiological patterns that are consistent with known sex-specific adaptations to endurance training.

Regarding the lipid metabolism, females exhibited higher HDL concentrations both pre- and post-camp, which may align with evidence that women rely more heavily on lipid metabolism during endurance exercise, partly mediated by estrogenic effects on substrate utilization [70], which may also show a more favorable basal cardiometabolic profile in our female endurance participants.

In contrast, males demonstrated higher creatinine and uric acid pre- and post-camp, reflecting their greater basal skeletal muscle mass. In addition, the hematological parameters RBC, hemoglobin, and hematocrit were all higher in males. This is not surprising, as norm values for these parameters are higher in men [71]. Although a sex-specific difference in erythropoiesis is possible due to different androgenic levels in testosterone [72], it is rather unlikely to occur during a short training time of one week, especially without the additional effect of high altitudes or other hypoxic factors. Additionally, the sex-specific change in MCHC suggests differential hematological responses, possibly related to fluid balance or the first signs of erythrocyte turnover during intensive training. Interestingly, females showed higher baseline ferritin levels, which contrasts to the general norm values in the general population, but may reflect the increased training effort, supplementation, or acute inflammatory response compared to the male participants.

The observed increase in LDH exclusively in females may indicate greater exercise-induced metabolic or muscle-related stress by the training camp. Here, the reason can only be speculated: either the individual regeneration time between the training sessions for the female participants was too short, or they generally trained harder than their male counterparts.

Overall, these findings may hint to possible different acute adaptations to the training camp, but mechanistically, this can only be speculated. Here, the sample size increases with additional specific examinations, and the assessment of sex hormones and the androgenic response has to be performed. Furthermore, the menstrual cycle was not assessed, which may particularly influence the hormonal and hematological parameters.

### 4.7. Limitations

This study has several limitations. First, the absence of a control group limits the ability to attribute observed changes solely to the training camp. Although the before–during–after design provides an internal comparison, seasonal training progression or environmental factors may have contributed.

Second, the training load was self-selected and not standardized. Participants were free to join or skip sessions, and differences in individual training volume, intensity distribution, and prior training history may have influenced the results.

Third, post-camp measurement timing was performed eight days after the training camp to allow for partial recovery. While this reduces acute exercise effects, it may have obscured peak responses of biomarkers such as VEGF, CK, or inflammatory cytokines, which show rapid kinetics. Thus, acute effects might have been missed, and mechanistic interpretation should be regarded with caution.

Fourth, dietary intake was self-reported via an app-based food diary, which is susceptible to recall and reporting bias. Macronutrient and caloric estimations must therefore be interpreted cautiously.

Fifth, the study cohort was heterogeneous regarding age, sex, sport background (running vs cycling), and baseline fitness, potentially influencing training adaptations and biomarker responses.

Finally, while circulating markers of mitochondrial biogenesis (e.g., PGC1-α, mtDNA) were assessed, direct measurements of mitochondrial content or function in muscle tissue (e.g., biopsy-based citrate synthase activity, OXPHOS protein quantification) were not feasible, limiting mechanistic interpretation.

## 5. Conclusions

In recreational athletes, a one-week endurance training camp in a holiday-like setting was associated with distinct changes in biomarkers related to systemic stress regulation and cellular stress responses, while sleep quality and subjective mental performance/stress remained stable or improved. In contrast, lactate-derived endurance performance outcomes did not improve, as velocities at fixed lactate concentrations (2–4 mmol/L) and threshold velocities remained unchanged and VO_2_max showed no statistically significant change. Together, these findings suggest that short training camps may primarily elicit short-term biological and stress-regulatory responses, whereas measurable performance adaptations require longer duration and/or more targeted training prescriptions. The concurrent rise in muscle-/cell-stress markers underscores the importance of individualized load management and adequate recovery when recreational athletes markedly increase their training volume.

## Figures and Tables

**Table 1:** Anthropometrics of the study participants before (pre) and after (post) the 1-week training camp. BMI: body mass index; SMM: skeletal muscle mass. Significance set at *p* ≤ 0.05. All values are given as mean ± standard deviation.

N = 35 [18 Male/17 Female]	Pre [n = 35]	Post [n = 27]	*p*-Value
**Age [years]**	46.4 ± 14.0		
**Height [cm]**	173.6 ± 10.6		
**Body mass [kg]**	70.34 ± 14.69	70.24 ± 15.56	**0.0306**
**BMI [kg/m^2^]**	23.21 ± 3.30	23.16 ± 3.51	**0.0174**
**Phase angle [°]**	5.76 ± 0.65	5.97 ± 0.68	**0.0003**
**SMM [kg]**	31.87 ± 6.95	32.34 ± 7.47	0.5000
**Body fat mass [kg]**	13.46 ± 7.19	12.54 ± 7.09	**0.0366**
**Body fat [%]**	18.84 ± 7.46	17.56 ± 7.62	**0.0370**

**Table 2:** Performance characteristics determined by lactate diagnostics before (pre) and after (post) the 1-week training camp. LT: lactate threshold; IAT: individual anaerobic threshold; Lac_Xmmol_: velocity at the respective lactate concentration of 2, 3 or 4 mmol; Lac_max_: point of maximal measured lactate; VO_2_max: calculated maximal oxygen capacity per body mass; HR: heart rate. Significance set at *p* ≤ 0.05. All values are given as mean ± standard deviation.

	Pre [n = 35]	Post [n = 34]	*p*-Value
**Lactate at LT [mmol/L]**	1.12 ± 0.51	1.34 ± 0.64	**0.0193**
**Lactate at IAT [mmol/L]**	2.62 ± 0.52	2.84 ± 0.65	**0.0185**
**Velocity at Lac_2mmol_ [km/h]**	11.32 ± 1.68	11.18 ± 1.74	0.3428
**Velocity at Lac_3mmol_ [km/h]**	12.09 ± 1.78	12.12 ± 1.93	0.8078
**Velocity at Lac_4mmol_ [km/h]**	12.92 ± 1.77	13.08 ± 1.82	0.3777
**Velocity at Lac_max_ [km/h]**	0.439 ± 0.204	0.440 ± 0.187	0.9046
**VO_2_max [ml/min/kg]**	45.24 ± 6.57	46.37 ± 7.35	0.0551
**HR at IAT [beats/min]**	154.3 ± 16.4	160.5 ± 14.2	**0.0158**

## Data Availability

All data are available on reasonable request from the corresponding author.

## Supplementary Materials

The following supporting information can be downloaded at https://www.mdpi.com/article/10.3390/sports14050200/s1, Figure S1: (A) Experimental design of the WAVE study, (B): Participant flow chart; Figure S2: Macronutrient distribution and number of exercise sessions in the week before (pre), during (training camp) and in the week after (post) the training camp; Figure S3: Sleep quality in the week before (pre), during (training camp) and in the week after (post) the training camp; Figure S4: Descriptive development of the recovery and stress assessed with the SRSS questionnaire; Figure S5: Blood cell markers before (pre) and after (post) the training camp; Figure S6: Lipid and sugar markers before (pre) and after (post) the training camp; Figure S7: Further plasma variables glomerular filtration rate (GFR), vitamin D and thyroid-stimulating hormone (TSH) before (pre) and after (post) the training camp. Table S1: Mean percental difference of Short Recovery and Stress Scale (SRSS) items during the study duration; File S1: Training Endurance Camp + WAVE-study Mallorca 2025: Schedule Overview; File S2: Sex stratified analysis of the WAVE-study participants (descriptive only).

## Abbreviations

The following abbreviations are used in this manuscript:

VEGF	vascular endothelial growth factor
GDF-15	growth differentiation factor 15
mtDNA	mitochondrial deoxyribonucleic acid
PGC1-alpha	peroxisome proliferator-activated receptor gamma coactivator 1-alpha
LDH	lactate dehydrogenase
VO2max	maximal oxygen capacity
LT	lactate at lactate threshold
IAT	lactate at the individual anaerobic threshold
Lacmax	velocity at maximum lactate
HR	heart rate
ASC	Ausdauer Sport Club
DGE	German Nutrition Society
FSVS	German version of the Athlete Sleep Behavior Questionnaire (ASBQ)
SRSS	Short Recovery and Stress Scale (German version)
STROBE	Strengthening the Reporting of Observational Studies in Epidemiology
CK	creatine kinase
AST	aspartate transaminase
ALT	alanine aminotransferase
GGT	gamma-glutamyltransferase
HDL	high-density lipoprotein
LDL	low-density lipoprotein
GFR	glomerular filtration rate
TSH	thyroid-stimulating hormone
B2M	nuclear gene
RT-qPCR	real-time qPCR
IFN-γ	interferon-gamma
CRP	C-reactive protein
ELISA	enzyme-linked immunosorbent assay
BMI	body mass index
SMM	skeletal muscle mass
UA	uric acid
RDW	relative distribution width
MCV	mean corpuscular volume
MCHC	mean corpuscular hemoglobin concentration
RBC	red blood cell
MCH	mean corpuscular hemoglobin
EA	energy availability
CHO	carbohydrate
RPE	rating of perceived exertion,
OXPHOS	oxidative phosphorylation
