# Supplementary material for: Physiological, Metabolic, and Mitochondrial Adaptations to a One-Week Endurance Training Camp in Recreational Athletes: An Observational Study
Source: Sports (Basel). 2026 May 13;14(5):200. doi: 10.3390/sports14050200 (PMC13211031; doi:10.3390/sports14050200)
Supplement: Supplementary file 1 [file sports-14-00200-s001.zip › Supplemental Figures and Table.pdf]

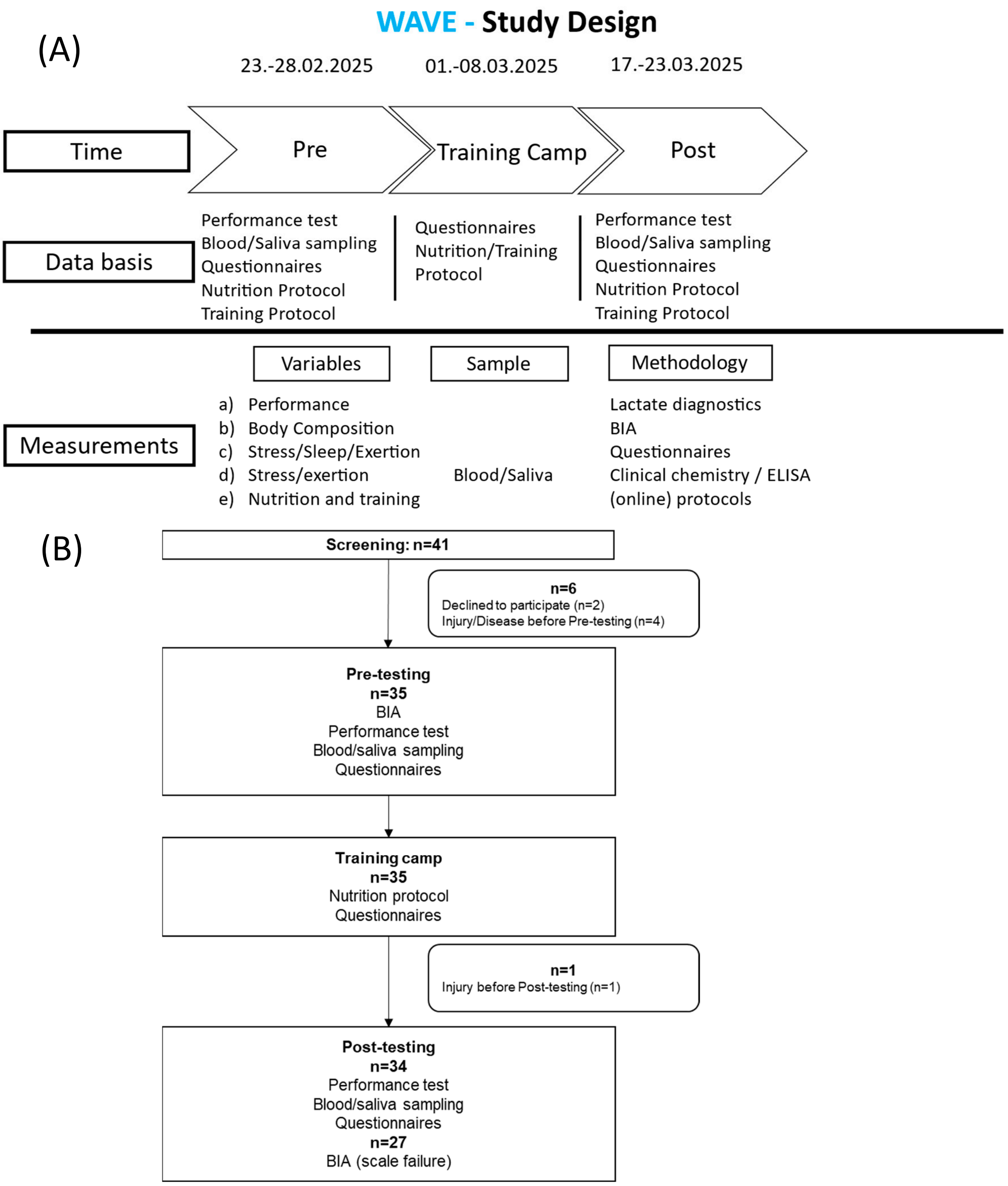

Figure S1. (A) Experimental design of the **WAVE**-study (**W**hat are the benefits of a week-long endurance training camp for recreational **a**thletes in terms of subjective and objecti**ve** performance and health parameters?). The experimental procedures consisted of performances testing, questionnaires, nutrition and training protocols as well as biosampling (blood/saliva) at three distinct time points. In the week before the training camp (pre), during the training camp (camp), and in the weeks following the training camp until the final performance testing (Post).

(B) Participant flow chart. From initially 41 screened participants, 6 dropped out due to injury/disease or organizational reasons. One further drop-out occurred due to

injury before post-testing. Seven body impedance analyses (BIA) could not be conducted due to measurement failures of the BIA scale at the end of the study.

https://doi.org/10.3390/xxxxx

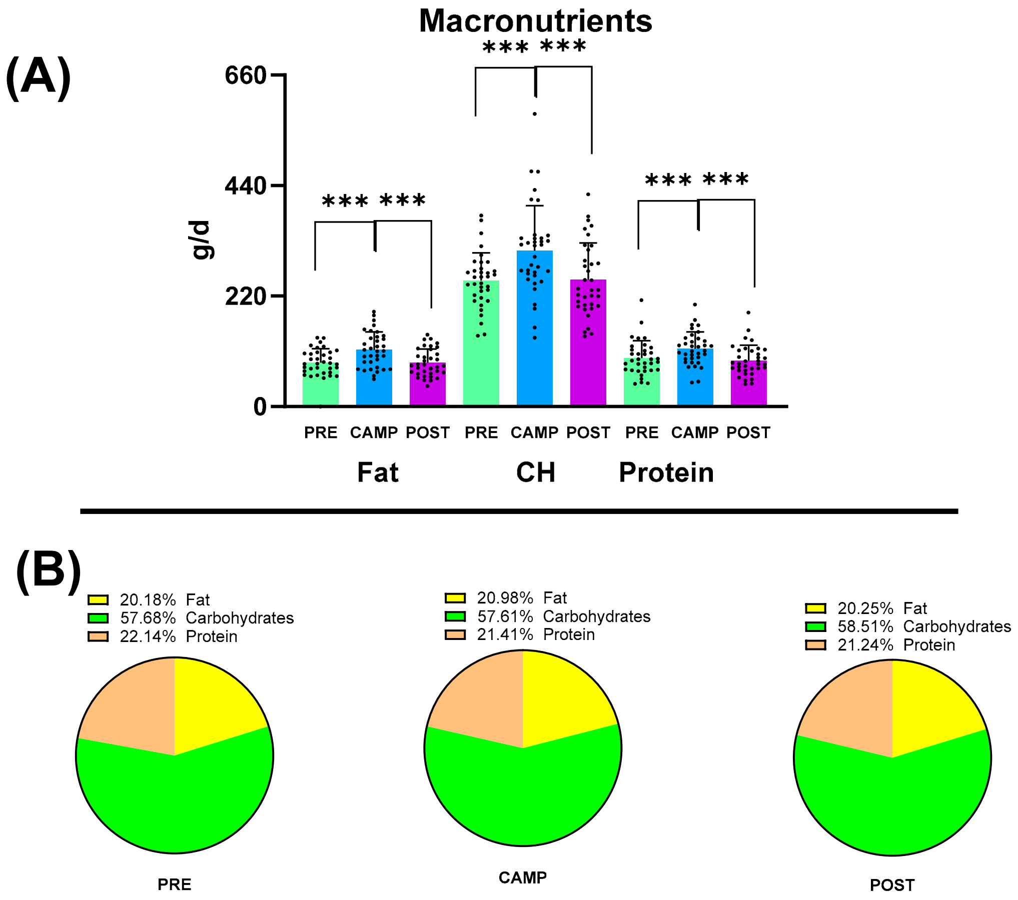

Figure S2. Macronutrient distribution in the week before (pre), during (training camp) and in the week after (post) the training camp. (A) Intake of all macronutrients fat, carbohydrates (CH) and protein increased during the camp, but decreased post. (B) Although general intake of fat, CH and protein increased during the training camp, the distribution of macronutrients remained unaltered in the diet of the endurance athletes. ***$p \leq 0.001$.

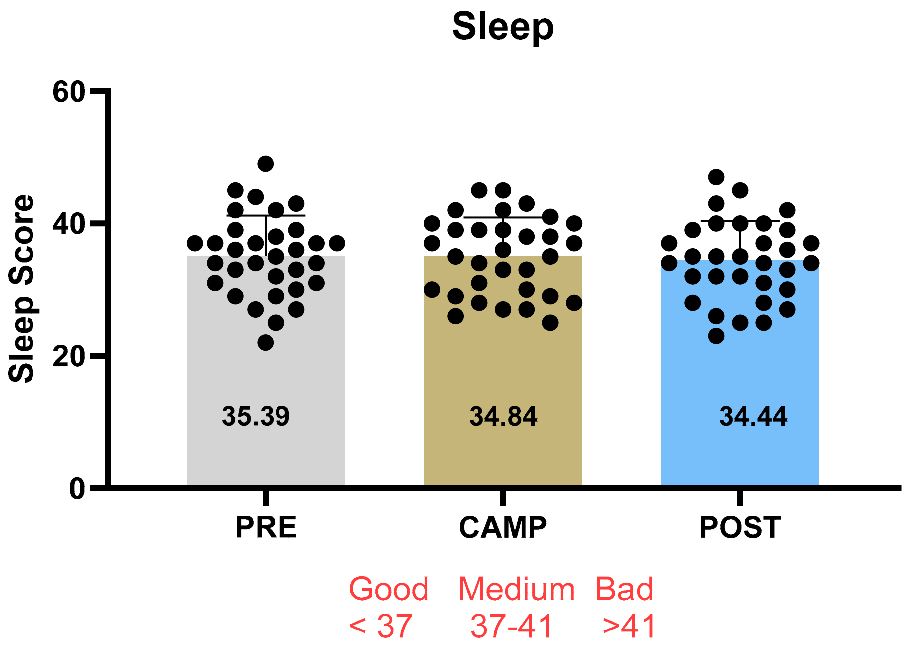

Figure S3. Sleep quality in the week before (pre), during (training camp) and in the week after (post) the training camp. Sleep quality scores did not change significantly during the whole study period (n=35).

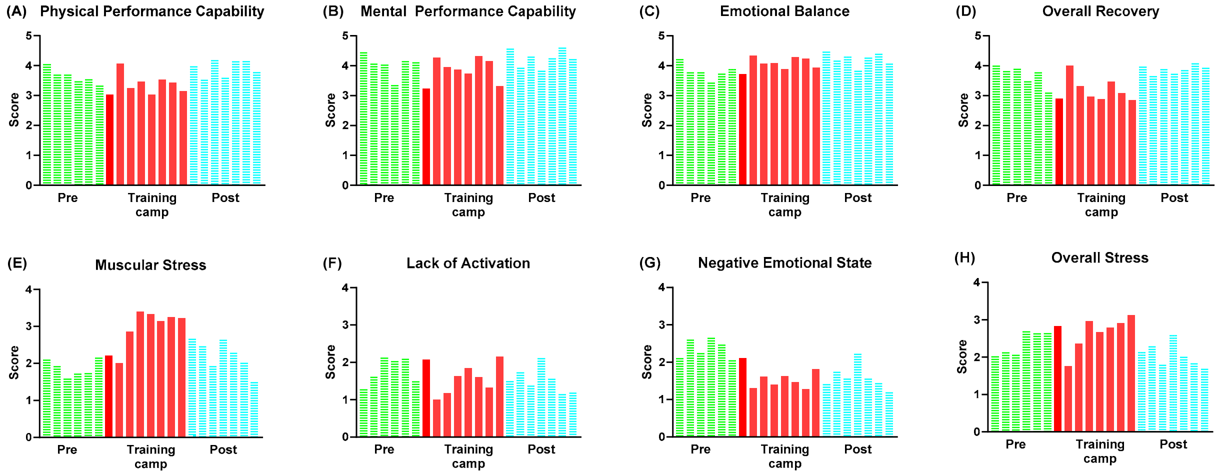

Figure S4. Descriptive development of the recovery and stress assessed with the SRSS questionnaire. Although (A) Physical Performance Capability and (D) Overall Recovery decreased with a concomitant increase in (E) Muscular Stress during the training camp, (B) Mental Performance Capability and (C) Emotional Balance remained stable over the study period while (G) the Negative Emotional State decreased during the camp compared to pre (n=35).

Table S1: Mean percental difference of Short Recovery and Stress Scale (SRSS) items during the study duration. Comparisons were performed between the values of the week before the camp and the training camp (Pre-Camp), between the camp and the week after the camp (Camp-Post) as well as between Pre and Post camp (Pre-Post).

SRSS item	Pre-Camp [%]	Camp-Post [%]	Pre-Post [%]
Physical performance capability	-8.0	16.7	7.4
Mental performance capability	-5.0	10.8	5.2
Emotional Balance	6.0	4.5	10.7
Overall recovery	-14.3	22.7	5.2
Muscular stress	53.8	-23.6	17.5
Lack of activation	-11.0	-3.5	-14.1
Negative emotional balance	-33.8	2.515274	-32.2
Overall stress	12.3	-22.6	-13.1

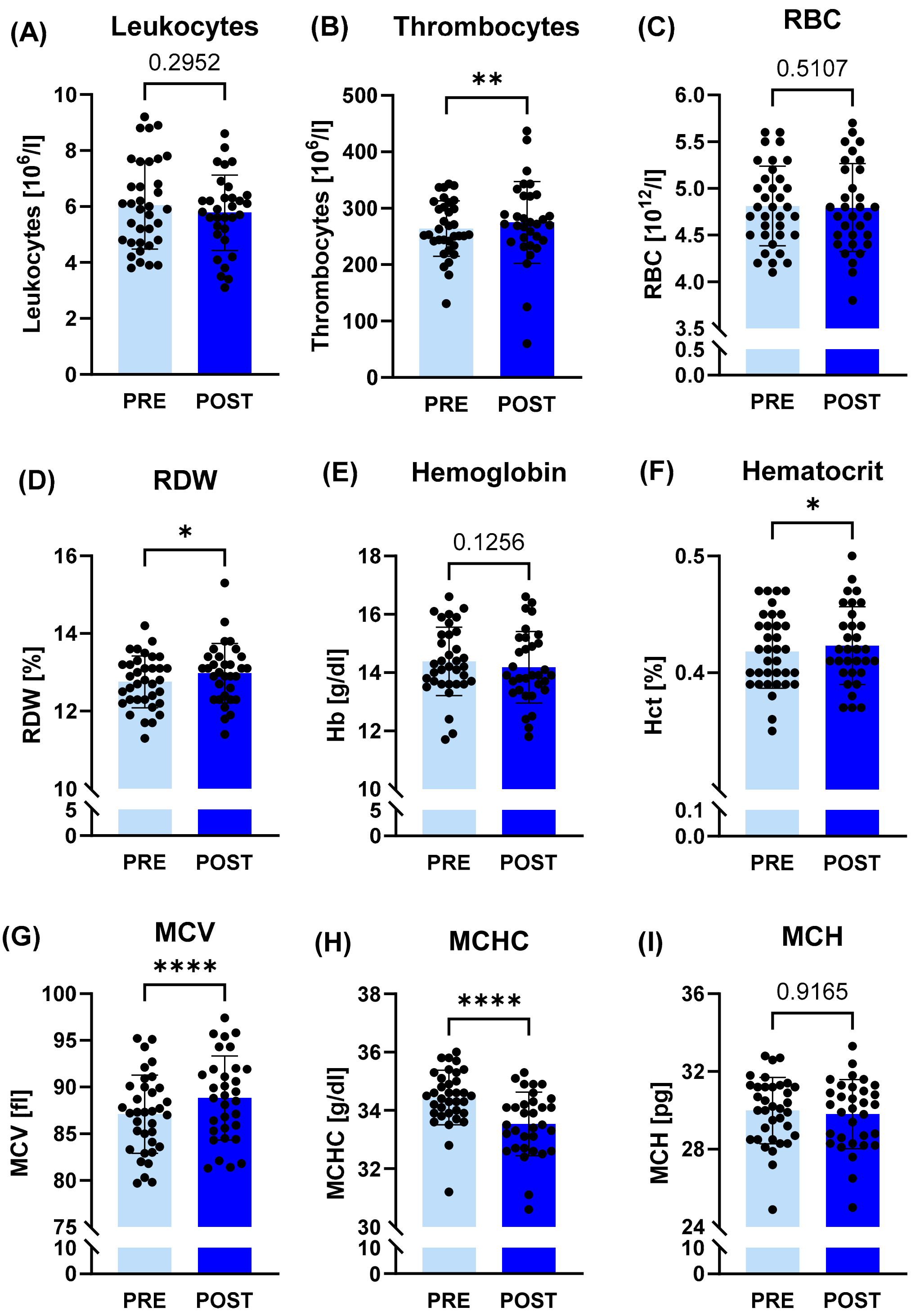

Figure S5. Blood cell markers before (pre) and after (post) the training camp. (A) Leukocytes, (C) red blood cells (RBC), (E) hemoglobin and (I) mean corpuscular hemoglobin (MCH) did not differ post compared to pre, whereas (B) thrombocyte concentrations, (D) relative distribution width (RDW), (F) hematocrit and (G) mean corpuscular volume (MCV) increased. Decreased values could be observed for (H) mean corpuscular hemoglobin concentration (MCHC). *$p \leq 0.05$ **$p \leq 0.01$; ****$p \leq 0.0001$.

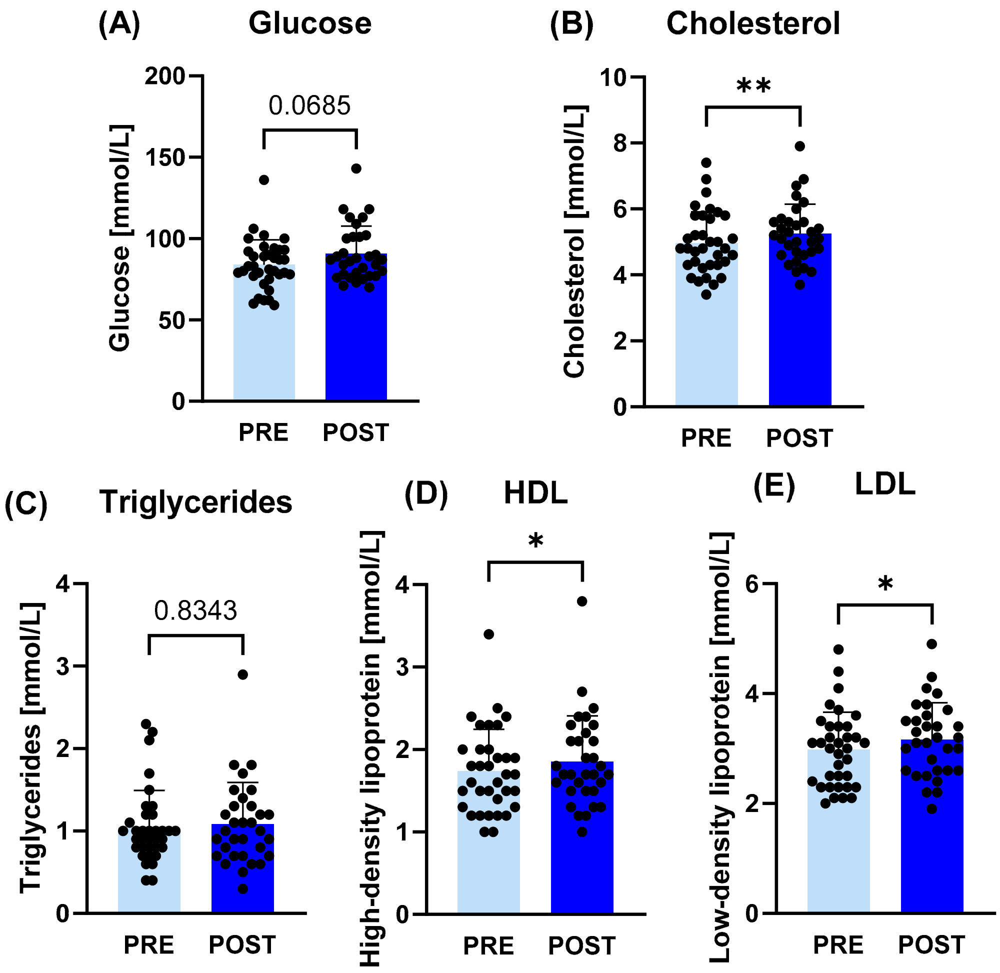

Figure S6. Lipid and sugar markers before (pre) and after (post) the training camp. (A) Glucose and (C) triglyceride levels did not differ at post compared to pre, whereas (B) cholesterol, (D) high density lipoprotein (HDL) and (E) low density lipoprotein (LDL) increased post training camp. *$p \leq 0.05$ **$p \leq 0.01$.

https://doi.org/10.3390/xxxxx

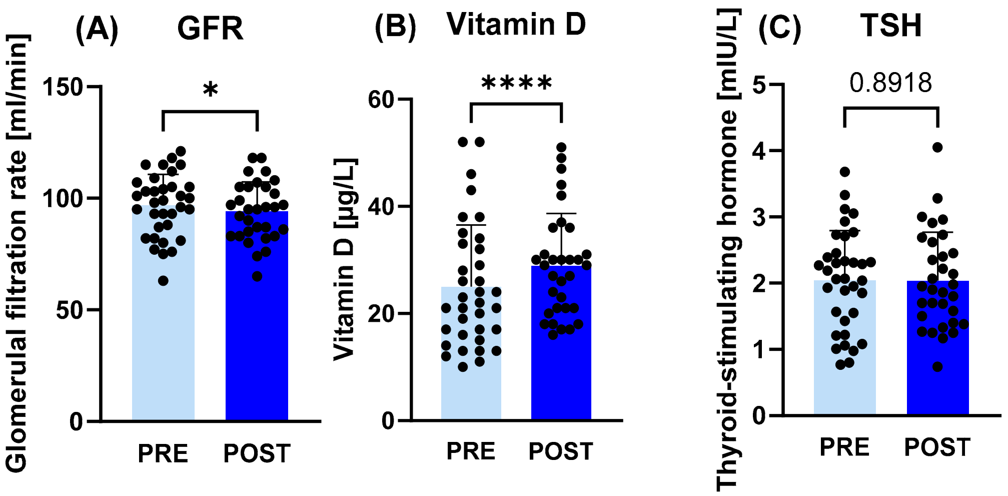

Figure S7. Further plasma variables glomerular filtration rate (GFR), vitamin D and thyroid-stimulating hormone (TSH) before (pre) and after (post) the training camp. (A) GFR decreased significantly after the training camp, while (B) vitamin D increased. There was no difference in (C) TSH between measurements at pre and post. $^{*}p \leq 0.05$ $^{****}p \leq 0.0001$.
