# Supplementary material for: Physiological, Metabolic, and Mitochondrial Adaptations to a One-Week Endurance Training Camp in Recreational Athletes: An Observational Study
Source: Sports (Basel). 2026 May 13;14(5):200. doi: 10.3390/sports14050200 (PMC13211031; doi:10.3390/sports14050200)
Supplement: Supplementary file 1 [file sports-14-00200-s001.zip › Supplementary File S1.pdf]

Training Endurance Camp + WAVE-study Mallorca 2025: Schedule Overview

Day	Sat 1.3.2025	Sun 2.3.2025	Mon 3.3.2025	Tue 4.3.2025	Wed 5.3.2025	Thu 6.3.2025	Fri 7.3.2025
	Arrival	- **Yoga**<br>- **Swimming** (optional)	- **Coordination, Mobility**<br>- **Swimming** (optional)	- **Stability training**<br>- **Swimming** (optional)	- Warm-up, **coordination**<br>- **Swimming** (optional)	- **Yoga**<br>- **Swimming** (optional)	- **Coordination, Mobility**<br>- **Swimming** (optional)
**Breakfast (from 8:00)**							
**Morning**	-	**Running**: Threshold Run<br>**Cycling**:<br>Group 1: 116 km / 1,110 EG<br>Group 2: 113 km / 1,150 EG<br>Group 3: 90 km / 500 EG	**Running:** Low intensity, coordination<br>**Cycling**:<br>Group 1: 115 km / 1,200 EG<br>Group 2: 113 km / 1,050 EG<br>Group 3: 90 km / 500 EG	**Cycling**:<br>Group 1:110 km / 1,800 EG<br>Group 2: 90 km / 1,200 EG<br>Group 3: 60 km / 1,000 EG	**Running**: Intervals<br>Cycling:<br>Group 1-3: 90-120 km / 300-500 EG	Aqua-jogging<br>**Cycling**:<br>Group 1: 115 km / 2,200 EG<br>Group 2: 90 km / 1.250 EG<br>Group 3: 70 km / 900 EG	**Running**: low intensity + 5 km race, coordination<br>**Cycling**:<br>Group 1-2: 125 km / 1,000 EG<br>Group 3: 80 km / 600 EG
**Afternoon**	**Running**: Exploratory run<br>Cycling: 60 km/ 300 EG	Aqua-jogging	**Running**: Fartlek	**Running**: Long run	**Running**: Mountain run		Mini-Endurance-Strength games
**From 16:00**	**Swimming** (optional)	**Swimming** (optional)	**Swimming** (optional)	**Swimming** (optional)	**Swimming** (optional)	**Swimming** (optional)	**Swimming** (optional)
**Forenoon (From 17:30)**	Introduction of trainers and participants	Daily review and preview	Daily review and preview	Yoga	Daily review and preview	Daily review and preview	
**Evening Dinner (from 18:30)**							
**Evening**			Lecture: “Nutrition”		Lecture: “Training for Marathon + Half Marathon”		Final review

Groups and Distances: Group 1: Longest distances and highest elevation / **Group 2**: Medium distances and elevation / **Group 3**: Shortest distances and lowest elevation
