# Supplementary material for: Physiological, Metabolic, and Mitochondrial Adaptations to a One-Week Endurance Training Camp in Recreational Athletes: An Observational Study
Source: Sports (Basel). 2026 May 13;14(5):200. doi: 10.3390/sports14050200 (PMC13211031; doi:10.3390/sports14050200)

Sex stratified analysis of the WAVE-study participants

The following pages include alternately the descriptive analysis of the laboratory markers as well as the respective graphical expression of the statistical analysis. w=women, m=men, Std.=Standard

**Parameter**	**GFR**			
**Sex**	**w**		**m**	
	**Pre**	**Post**	**Pre**	**Post**
Minimum	63.0	65.0	75.0	76.0
25% Percentile	84.5	83.0	84.5	86.0
Median	103.0	92.0	96.0	95.0
75% Percentile	115.0	111.0	102.0	99.0
Maximum	121.0	118.0	109.0	107.0
Range	58.0	53.0	34.0	31.0
Mean	99.5	94.9	93.9	93.4
Std. Deviation	16.4	16.5	10.9	8.7
Std. Error of Mean	4.0	4.1	2.6	2.3
	**Vitamin D**			
Minimum	11.0	17.0	10.0	16.0
25% Percentile	18.0	23.8	13.0	18.5
Median	24.0	30.0	21.0	25.5
75% Percentile	40.5	43.5	28.3	30.0
Maximum	52.0	51.0	38.0	37.0
Range	41.0	34.0	28.0	21.0
Mean	28.7	33.0	21.6	24.9
Std. Deviation	13.4	11.0	8.4	6.3
Std. Error of Mean	3.3	2.8	2.0	1.6
	**TSH**			
Minimum	1.0	1.3	0.8	0.7
25% Percentile	1.5	1.4	1.2	1.4
Median	2.3	1.9	2.1	1.9
75% Percentile	2.7	2.9	2.4	2.4
Maximum	3.3	4.1	3.7	2.9
Range	2.4	2.8	2.9	2.2
Mean	2.1	2.2	2.0	1.9
Std. Deviation	0.7	0.8	0.8	0.6
Std. Error of Mean	0.2	0.2	0.2	0.2

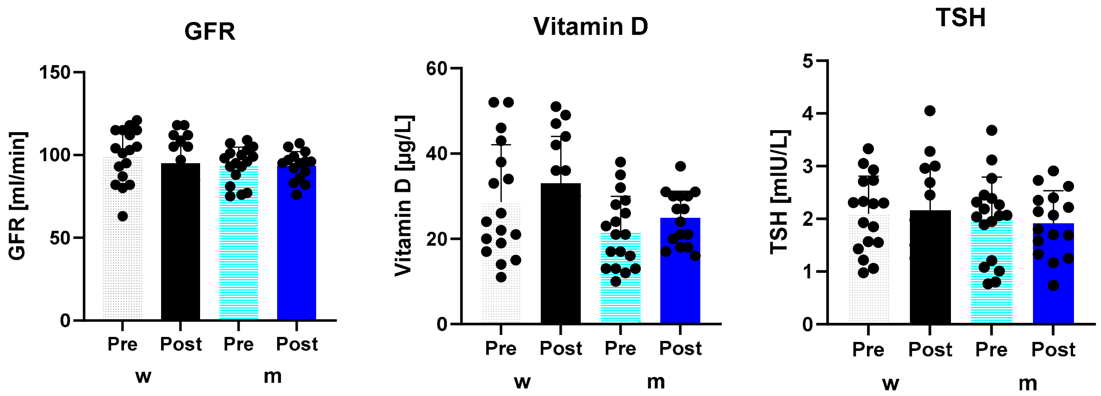

Parameter	Cortisol			
Sex	w		m	
	Pre	Post	Pre	Post
Minimum	0.1	0.1	0.1	0.1
25% Percentile	0.2	0.1	0.2	0.1
Median	0.3	0.2	0.2	0.2
75% Percentile	0.4	0.4	0.5	0.3
Maximum	0.7	0.6	0.6	0.8
Range	0.6	0.5	0.5	0.8
Mean	0.3	0.3	0.3	0.2
Std. Deviation	0.2	0.2	0.2	0.2
Std. Error of Mean	0.0	0.0	0.0	0.0
	**CRP**			
Minimum	64.2	47.6	80.3	83.8
25% Percentile	73.4	112.3	101.1	107.6
Median	191.7	233.1	157.0	213.9
75% Percentile	574.2	611.0	373.5	455.2
Maximum	2.823.0	2.103.0	958.7	745.1
Range	2.759.0	2.056.0	878.4	661.3
Mean	505.0	478.5	288.5	286.2
Std. Deviation	754.9	590.1	298.4	222.5
Std. Error of Mean	183.1	147.5	70.3	57.5
	**Uric Acid**			
Minimum	1.1	1.1	2.3	0.6
25% Percentile	3.2	2.7	3.1	2.6
Median	4.1	3.7	3.8	3.6
75% Percentile	4.7	4.8	5.4	4.7
Maximum	7.1	6.7	8.1	6.1
Range	5.9	5.6	5.8	5.5
Mean	4.1	3.8	4.3	3.5
Std. Deviation	1.5	1.5	1.7	1.5
Std. Error of Mean	0.4	0.4	0.4	0.4

	Urea			
Minimum	2.0	3.2	3.6	4.3
25% Percentile	3.9	4.3	5.0	5.4
Median	4.8	5.2	5.9	5.9
75% Percentile	5.9	6.0	6.4	6.7
Maximum	8.7	7.9	7.6	8.0
Range	6.7	4.7	4.0	3.7
Mean	4.9	5.2	5.7	6.0
Std. Deviation	1.7	1.3	1.1	1.0
Std. Error of Mean	0.4	0.3	0.3	0.3
	**Creatinine**			
Minimum	52.0	55.0	64.0	62.0
25% Percentile	58.0	61.3	73.5	75.3
Median	64.0	69.0	80.5	80.5
75% Percentile	70.5	73.3	89.5	87.8
Maximum	88.0	86.0	95.0	105.0
Range	36.0	31.0	31.0	43.0
Mean	65.5	68.9	81.3	82.1
Std. Deviation	10.3	9.6	9.1	10.4
Std. Error of Mean	2.5	2.4	2.1	2.6
	**Uric Acid**			
Minimum	133.0	158.0	246.0	254.0
25% Percentile	190.5	200.0	281.3	285.0
Median	216.0	234.5	312.5	300.5
75% Percentile	265.5	271.3	351.5	342.5
Maximum	322.0	312.0	412.0	390.0
Range	189.0	154.0	166.0	136.0
Mean	225.4	231.4	317.0	311.8
Std. Deviation	49.1	46.2	46.3	40.6
Std. Error of Mean	11.9	11.5	10.9	10.2
	**Bilirubin**			
Minimum	3.0	3.0	6.0	5.0
25% Percentile	4.0	4.3	7.0	7.0
Median	6.0	5.0	8.0	9.5
75% Percentile	9.5	8.8	11.3	11.0
Maximum	35.0	30.0	16.0	17.0
Range	32.0	27.0	10.0	12.0
Mean	8.8	7.4	9.4	9.4
Std. Deviation	9.0	6.4	3.1	3.3
Std. Error of Mean	2.2	1.6	0.7	0.8
	**AST**			
Minimum	19.0	20.0	19.0	20.0
25% Percentile	22.0	22.3	22.8	22.0

Median	25.0	27.5	26.5	27.0
75% Percentile	31.0	30.8	30.5	30.0
Maximum	38.0	50.0	34.0	52.0
Range	19.0	30.0	15.0	32.0
Mean	26.4	28.7	26.9	28.5
Std. Deviation	5.3	8.5	4.7	9.0
Std. Error of Mean	1.3	2.1	1.1	2.3
	**ALT**			
Minimum	12.0	14.0	15.0	14.0
25% Percentile	14.5	16.3	19.3	20.3
Median	18.0	19.5	22.0	24.5
75% Percentile	21.0	25.0	30.3	31.5
Maximum	50.0	101.0	44.0	47.0
Range	38.0	87.0	29.0	33.0
Mean	19.9	26.9	24.7	26.2
Std. Deviation	9.0	21.6	7.7	9.7
Std. Error of Mean	2.2	5.4	1.8	2.4
	**GGT**			
Minimum	8.0	11.0	12.0	13.0
25% Percentile	9.0	15.0	15.0	14.0
Median	15.0	16.0	19.0	20.0
75% Percentile	23.0	20.0	32.0	25.8
Maximum	111.0	139.0	58.0	57.0
Range	103.0	128.0	46.0	44.0
Mean	24.1	26.5	23.9	23.5
Std. Deviation	26.5	31.5	12.5	13.3
Std. Error of Mean	6.4	7.9	3.0	3.3
	**Ferritin**			
Minimum	8.0	2.7	45.0	34.0
25% Percentile	26.0	18.8	66.8	56.3
Median	46.0	31.5	124.0	96.5
75% Percentile	103.0	95.3	194.3	146.8
Maximum	296.0	294.0	475.0	463.0
Range	288.0	291.3	430.0	429.0
Mean	72.0	61.7	150.1	123.7
Std. Deviation	72.2	68.9	110.8	108.2
Std. Error of Mean	16.6	16.2	27.7	28.9
	**CK**			
Minimum	52.0	54.0	48.0	54.0
25% Percentile	88.0	95.8	117.0	108.3
Median	136.0	126.0	136.5	151.0
75% Percentile	165.0	220.5	162.5	235.0
Maximum	240.0	516.0	341.0	704.0

Range	188.0	462.0	293.0	650.0
Mean	133.7	174.3	152.9	202.1
Std. Deviation	52.9	129.9	68.1	164.5
Std. Error of Mean	12.8	32.5	16.1	41.1
	**Kynurenine**			
Minimum	1.9	1.7	2.3	1.9
25% Percentile	2.8	2.4	3.1	2.4
Median	3.4	2.9	3.8	2.8
75% Percentile	3.9	3.3	4.7	3.2
Maximum	4.9	3.6	4.8	3.7
Range	3.1	1.9	2.5	1.8
Mean	3.4	2.8	3.8	2.8
Std. Deviation	0.7	0.6	0.8	0.5
Std. Error of Mean	0.2	0.1	0.2	0.1
	**Interferon-γ**			
Minimum	86.0	73.8	91.2	74.6
25% Percentile	91.4	75.2	93.3	76.3
Median	93.4	76.4	98.5	80.6
75% Percentile	109.9	85.6	111.0	82.5
Maximum	148.9	97.6	201.0	104.8
Range	62.9	23.8	109.8	30.2
Mean	101.4	79.7	107.8	82.7
Std. Deviation	17.0	6.9	27.9	9.2
Std. Error of Mean	4.1	1.7	6.6	2.3
	**Klotho**			
Minimum	0.1	0.2	0.1	0.2
25% Percentile	0.1	0.2	0.1	0.2
Median	0.2	0.2	0.2	0.2
75% Percentile	0.2	0.2	0.2	0.2
Maximum	0.2	0.3	0.2	0.3
Range	0.0	0.1	0.0	0.1
Mean	0.2	0.2	0.2	0.2
Std. Deviation	0.0	0.0	0.0	0.0
Std. Error of Mean	0.0	0.0	0.0	0.0
	**LDH**			
Minimum	1.735.0	1.031.0	1.732.0	1.467.0
25% Percentile	1.965.0	2.451.0	1.910.0	2.020.0
Median	2.507.0	3.891.0	2.163.0	2.596.0
75% Percentile	2.630.0	4.312.0	2.623.0	3.612.0
Maximum	3.320.0	6.465.0	3.064.0	4.985.0
Range	1.586.0	5.434.0	1.332.0	3.518.0
Mean	2.439.0	3.474.0	2.291.0	2.815.0

Std. Deviation	503.9	1.386.0	436.2	1.045.0
Std. Error of Mean	122.2	346.5	102.8	261.3

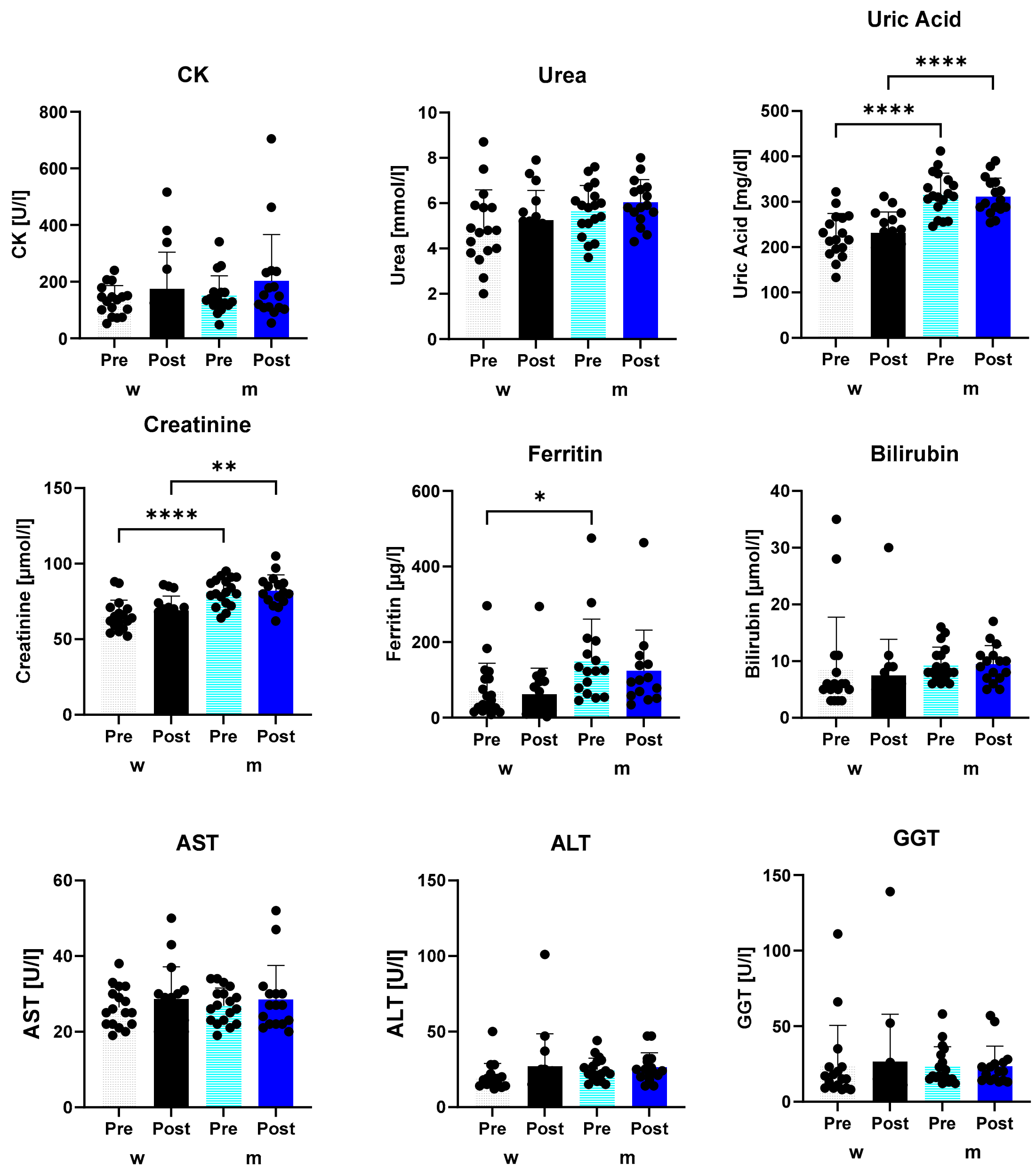

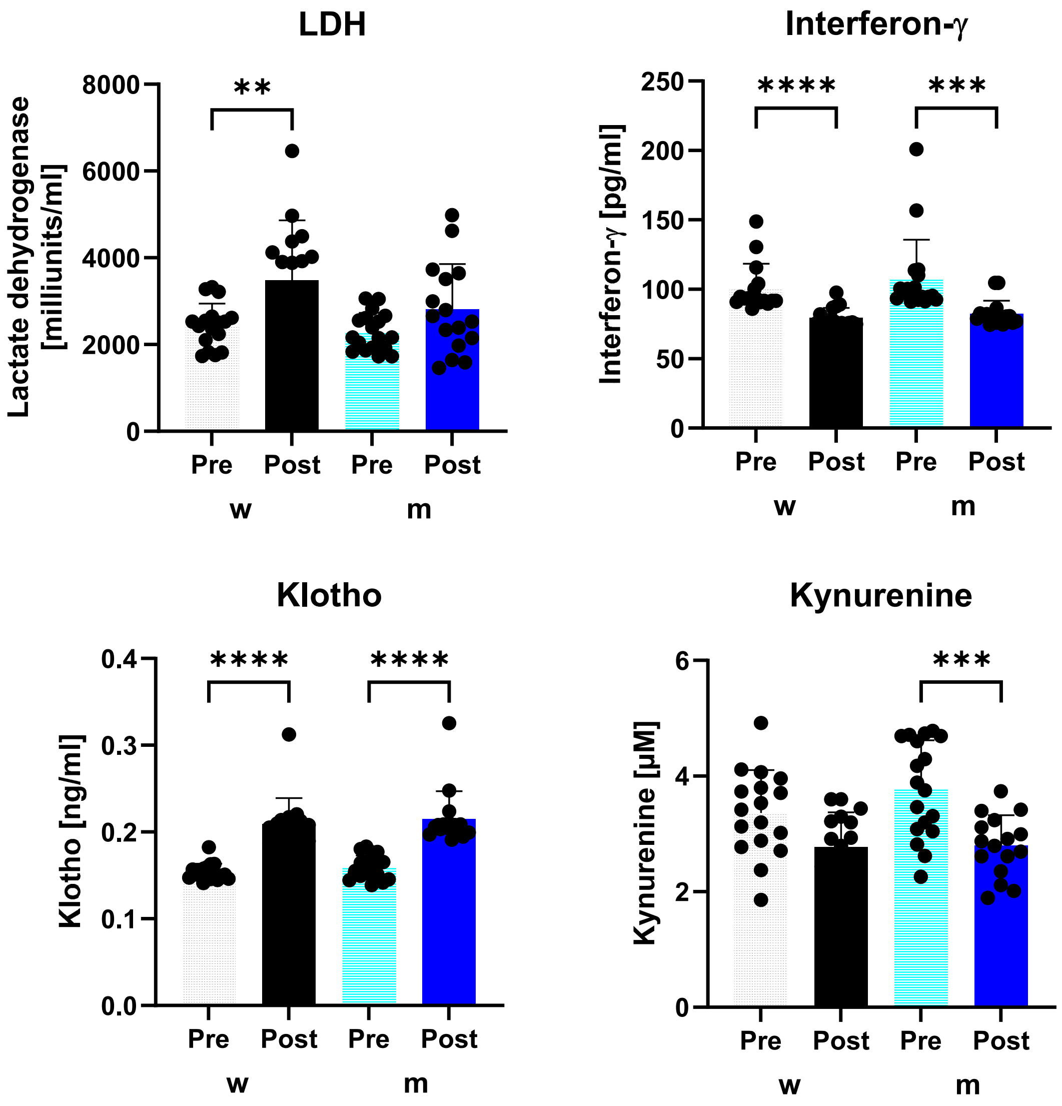

Parameter	Triglycerides			
Sex	w		m	
	Pre	Post	Pre	Post
Minimum	0.6	0.5	0.4	0.3
25% Percentile	0.8	0.7	0.9	0.8
Median	0.9	0.9	1.0	1.1
75% Percentile	1.0	1.3	1.4	1.4
Maximum	1.7	1.8	2.3	2.9
Range	1.1	1.3	1.9	2.6
Mean	0.9	1.0	1.2	1.2
Std. Deviation	0.3	0.4	0.5	0.6
Std. Error of Mean	0.1	0.1	0.1	0.2
	LDL			
Minimum	2.0	2.2	2.1	1.9
25% Percentile	2.3	2.5	2.7	2.9
Median	2.5	3.0	3.1	3.3

75% Percentile	3.5	3.8	3.4	3.5
Maximum	4.8	4.9	3.8	4.0
Range	2.8	2.7	1.7	2.1
Mean	3.0	3.2	3.0	3.2
Std. Deviation	0.8	0.8	0.5	0.5
Std. Error of Mean	0.2	0.2	0.1	0.1
	**HDL**			
Minimum	1.2	1.2	1.0	1.0
25% Percentile	1.4	1.6	1.3	1.4
Median	2.0	2.2	1.5	1.7
75% Percentile	2.4	2.4	1.7	1.8
Maximum	3.4	3.8	2.3	2.1
Range	2.2	2.6	1.3	1.1
Mean	2.0	2.1	1.5	1.6
Std. Deviation	0.6	0.6	0.3	0.3
Std. Error of Mean	0.1	0.2	0.1	0.1
	**Cholesterol**			
Minimum				
25% Percentile	3.4	4.1	3.8	3.7
Median	4.3	4.7	4.3	4.6
75% Percentile	4.8	5.4	4.8	5.1
Maximum	6.0	6.3	5.2	5.4
Range	7.4	7.9	5.9	5.7
	4.0	3.8	2.1	2.0
Mean				
Std. Deviation	5.1	5.5	4.8	5.0
Std. Error of Mean	1.1	1.1	0.6	0.5
	0.3	0.3	0.1	0.1
	**Glucose**			
Minimum	59.0	70.0	60.0	71.0
25% Percentile	73.5	79.3	77.8	77.0
Median	80.0	87.5	87.5	86.5
75% Percentile	92.0	99.3	93.0	101.0
Maximum	136.0	143.0	106.0	118.0
Range	77.0	73.0	46.0	47.0
Mean	83.5	91.7	84.4	90.0
Std. Deviation	17.5	19.0	13.3	15.0
Std. Error of Mean	4.3	4.7	3.1	3.7

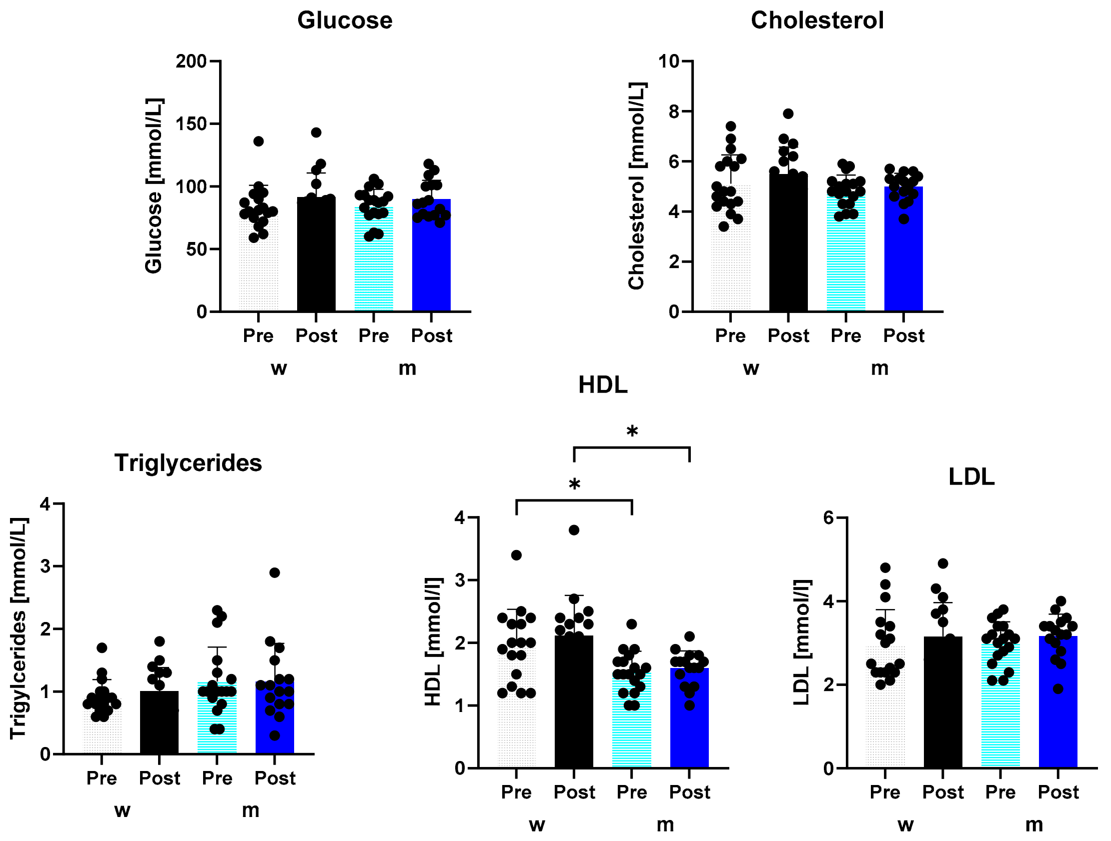

Parameter	GDF-15			
Sex	w		m	
	Pre	Post	Pre	Post
Minimum	60.2	62.1	71.2	54.8
25% Percentile	92.8	127.5	94.4	72.2
Median	149.7	148.5	151.2	94.0
75% Percentile	169.2	190.3	189.5	225.3
Maximum	209.6	286.4	281.9	298.5
Range	149.5	224.3	210.6	243.7
Mean	139.0	161.9	152.1	143.1
Std. Deviation	46.2	61.8	59.1	91.2
Std. Error of Mean	11.2	15.5	13.9	22.8
	VEGF			
Minimum	18.4	9.3	18.6	7.6
25% Percentile	24.7	11.7	20.4	9.3
Median	29.4	17.5	24.5	13.9
75% Percentile	39.8	19.8	28.0	17.0
Maximum	49.9	66.6	32.4	26.7
Range	31.4	57.2	13.8	19.1
Mean	31.4	19.5	24.7	14.2
Std. Deviation	8.9	13.8	4.7	5.3
Std. Error of Mean	2.2	3.4	1.1	1.3

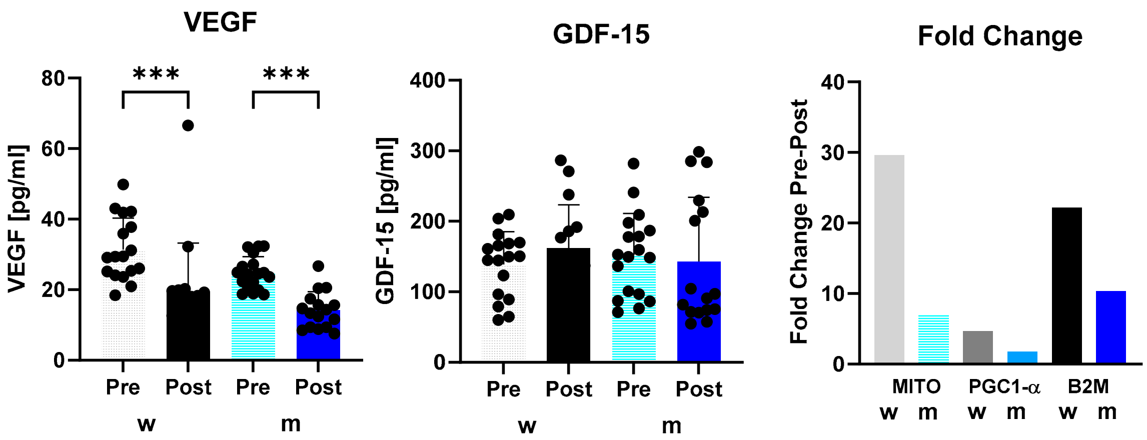

Parameter	Leukocytes			
Sex	w		m	
	Pre	Post	Pre	Post
Minimum	3.8	3.5	3.9	3.1
25% Percentile	4.7	4.9	4.8	5.4
Median	5.9	5.9	6.0	5.9
75% Percentile	6.7	6.3	7.6	6.6
Maximum	9.2	8.1	8.9	8.6
Range	5.4	4.6	5.0	5.5
Mean	5.9	5.7	6.2	5.8
Std. Deviation	1.6	1.2	1.6	1.5
Std. Error of Mean	0.4	0.3	0.4	0.4
	**RBC**			
Minimum	4.1	3.8	4.2	4.1
25% Percentile	4.3	4.3	4.8	4.5
Median	4.6	4.7	5.1	5.1
75% Percentile	4.8	4.8	5.4	5.5
Maximum	5.1	5.2	5.6	5.7
Range	1.0	1.4	1.4	1.6
Mean	4.6	4.6	5.1	5.0
Std. Deviation	0.3	0.3	0.4	0.5
Std. Error of Mean	0.1	0.1	0.1	0.1
	**Thrombocytes**			
Minimum	196.0	232.0	131.0	60.0
25% Percentile	251.0	269.3	218.5	220.0
Median	277.0	281.0	244.0	254.5
75% Percentile	306.0	338.3	291.5	313.0
Maximum	343.0	437.0	340.0	343.0
Range	147.0	205.0	209.0	283.0

Mean	277.1	301.7	250.8	248.1
Std. Deviation	37.0	60.9	56.8	75.0
Std. Error of Mean	9.0	15.2	13.8	18.7
	**MCH**			
Minimum	24.9	25.0	27.2	26.5
25% Percentile	28.6	28.4	28.5	28.4
Median	30.1	30.4	30.1	30.0
75% Percentile	31.3	31.2	31.2	31.2
Maximum	32.8	32.4	32.6	33.3
Range	7.9	7.4	5.4	6.8
Mean	30.0	29.7	30.0	29.9
Std. Deviation	2.0	1.9	1.5	1.8
Std. Error of Mean	0.5	0.5	0.4	0.4
	**MCHC**			
Minimum	31.2	30.6	33.6	32.5
25% Percentile	33.7	32.6	34.1	32.9
Median	34.3	33.3	34.6	34.2
75% Percentile	35.2	33.8	35.2	34.8
Maximum	36.0	34.9	35.8	35.3
Range	4.8	4.3	2.2	2.8
Mean	34.3	33.1	34.6	34.0
Std. Deviation	1.2	1.1	0.7	0.9
Std. Error of Mean	0.3	0.3	0.2	0.2
	**MCV**			
Minimum	79.8	81.8	79.7	81.3
25% Percentile	84.6	85.5	82.8	84.6
Median	87.3	89.2	87.4	88.1
75% Percentile	89.7	93.7	90.3	91.1
Maximum	95.2	97.4	94.3	95.4
Range	15.4	15.6	14.6	14.1
Mean	87.5	89.7	86.7	88.0
Std. Deviation	4.1	4.6	4.3	4.4
Std. Error of Mean	1.0	1.2	1.0	1.1
	**RDW**			
Minimum	11.3	11.4	11.7	11.9
25% Percentile	12.2	12.4	12.5	12.3
Median	12.5	13.1	12.9	13.0
75% Percentile	13.3	13.6	13.2	13.2
Maximum	14.2	15.3	13.6	13.8
Range	2.9	3.9	1.9	1.9
Mean	12.7	13.1	12.8	12.9
Std. Deviation	0.8	1.0	0.5	0.5
Std. Error of Mean	0.2	0.2	0.1	0.1

	Hemoglobin			
Minimum	11.7	11.8	13.6	13.3
25% Percentile	13.4	12.7	14.2	13.8
Median	13.7	13.5	15.3	15.1
75% Percentile	14.3	13.9	15.9	16.0
Maximum	14.8	14.9	16.6	16.6
Range	3.1	3.1	3.0	3.3
Mean	13.6	13.4	15.1	14.9
Std. Deviation	0.9	0.9	1.0	1.1
Std. Error of Mean	0.2	0.2	0.2	0.3
	**Hematocrit**			
Minimum	0.4	0.4	0.4	0.4
25% Percentile	0.4	0.4	0.4	0.4
Median	0.4	0.4	0.4	0.4
75% Percentile	0.4	0.4	0.5	0.5
Maximum	0.4	0.5	0.5	0.5
Range	0.1	0.1	0.1	0.1
Mean	0.4	0.4	0.4	0.4
Std. Deviation	0.0	0.0	0.0	0.0
Std. Error of Mean	0.0	0.0	0.0	0.0

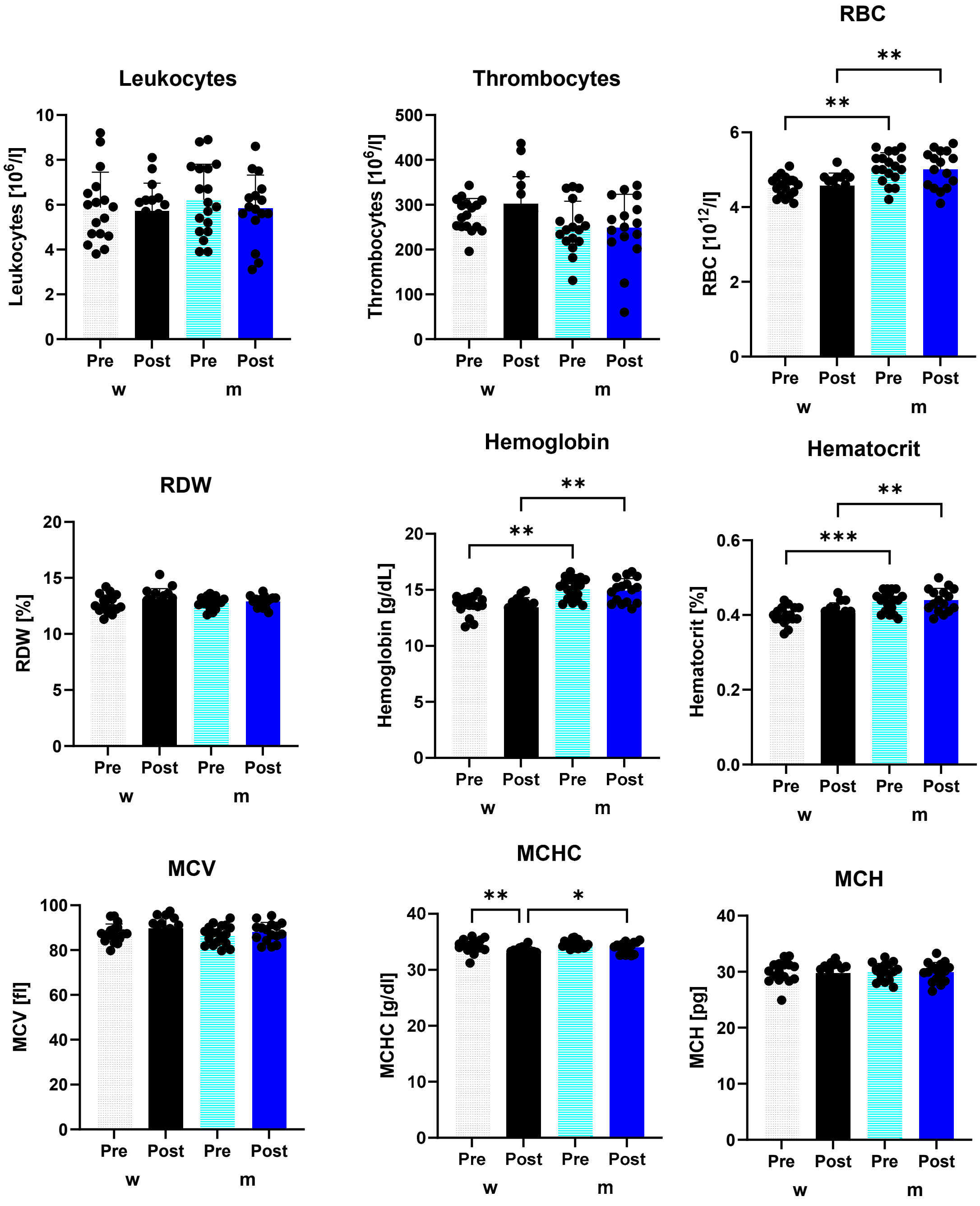
Leukocytes
Leukocytes [10^6/l]
0
2
4
6
8
10
Thrombocytes
Thrombocytes [10^6/l]
0
100
200
300
400
500
RBC
RBC [10^12/l]
0
2
4
6
**
**
RDW
RDW [%]
0
5
10
15
20
Hemoglobin
Hemoglobin [g/dL]
0
5
10
15
20
**
**
Hematocrit
Hematocrit [%]
0.0
0.2
0.4
0.6
***
**
MCV
MCV [fl]
0
20
40
60
80
100
MCHC
MCHC [g/dl]
0
10
20
30
40
**
*
MCH
MCH [pg]
0
10
20
30
40
Pre
Post
Pre
Post
w
m

Parameter	Sleep					
**Sex**	**w**			**m**		
	**Pre**	**Camp**	**Post**	**Pre**	**Camp**	**Post**
Minimum	22.0	26.0	23.0	25.0	25.0	25.0
25% Percentile	29.5	28.3	28.0	32.3	33.3	32.5
Median	34.0	32.0	32.5	36.5	37.0	36.5
75% Percentile	37.8	38.8	36.5	41.3	41.8	39.8
Maximum	45.0	42.0	43.0	49.0	45.0	47.0
Range	23.0	16.0	20.0	24.0	20.0	22.0
Mean	34.1	33.4	32.7	36.2	36.7	36.1
Std. Deviation	5.9	5.4	5.6	6.2	6.1	6.1
Std. Error of Mean	1.5	1.3	1.4	1.6	1.5	1.5

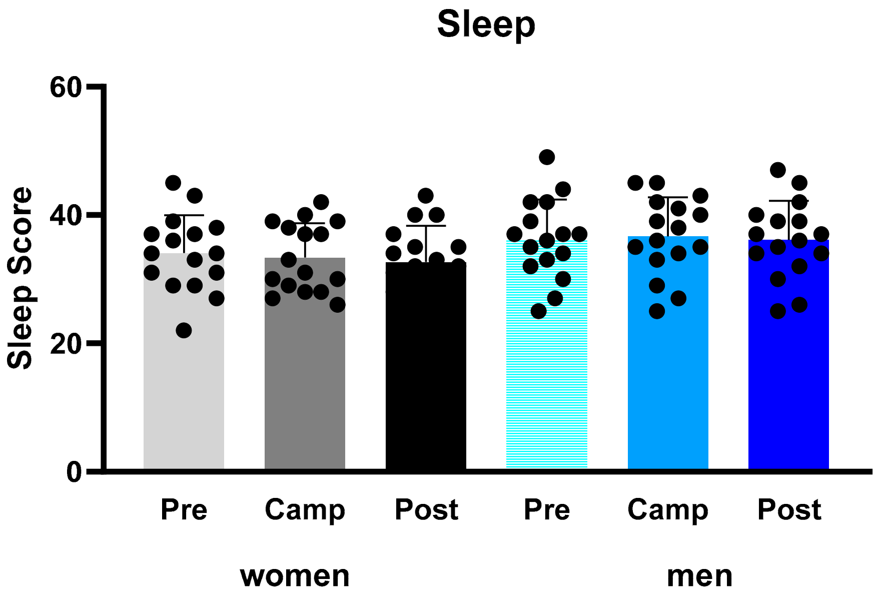

Supplement: Supplementary file 1 [file sports-14-00200-s001.zip › Supplementary File S2.pdf]
